# Increased encoding of aversive stimuli by lateral habenula neurons during Δ^9^-tetrahydrocannabinol withdrawal

**DOI:** 10.1038/s41467-026-77429-9

**Published:** 2026-09-03

**Authors:** Eun-Kyung Hwang, Deborah Daphne, Charlie J. Maddox, Agustin Zapata, Alexander F. Hoffman, Carl R. Lupica

**Affiliations:** 1 National Institutes of Health, National Institute on Drug Abuse Intramural Research Program, Cellular and Neurocomputational Systems Branch, Electrophysiology Research Section, Baltimore, MD USA; 2 The Lewis Katz School of Medicine, Center for Substance Abuse Research, Temple University, Philadelphia, PA USA; 3 Present Address: Department of Behavioral Neuroscience, Oregon Health & Sciences University, Portland, OR USA

**Keywords:** Cellular neuroscience, Neural circuits

## Abstract

The lateral habenula (LHb) integrates cortical and basal forebrain (BF) input and is implicated in negative affective states, but its involvement in negative affect associated with cannabis withdrawal is unknown. Here, effects of withdrawal from the psychoactive cannabis constituent, Δ^9^-tetrahydrocannabinol (Δ^9^-THC), on fear conditioning (FC) and LHb function were measured in rats using calcium photometry and electrophysiology. FC-associated LHb calcium signals, freezing behavior, and LHb excitatory-inhibitory (E-I) ratios were increased during Δ^9^-THC withdrawal, and local GABA release was impaired, indicating disinhibition of LHb output neurons. GABA release was also impaired at optogenetically activated BF-LHb axons, and not from ventral tegmental area-LHb inputs. The results indicate that Δ^9^-THC withdrawal increases LHb excitability through disinhibition, contributing to increased fear of primary and conditioned aversive stimuli. Our findings implicate the LHb in cannabis withdrawal-associated negative affect and offer insight into the neural mechanisms of cannabis use disorder.

## Abstract (web summary)

Withdrawal from extended cannabis use is associated with negative emotional states. Here, the authors show that withdrawal from the psychoactive cannabis molecule, THC, increased lateral habenula excitability and the strength of fear conditioning.

## Introduction

Addictive drug use is thought to be initially driven by the desire to experience pleasurable effects including euphoria, elevated mood, and a general state of well-being, via interactions with brain reward systems^1^. However, long-term drug use promotes adaptation of neural circuits leading to the development of dependence that is manifest as a withdrawal state when drug use ceases. Withdrawal from several drugs, including opioids, alcohol, psychostimulants, nicotine, and cannabis, is associated with both physiological and psychological symptoms that include increased sensitivity to stress, irritability, heightened anxiety, and a general negative dysphoric state^2^. Avoidance of negative withdrawal symptoms through continued use of the drug is also considered a primary obstacle to drug abstinence^1–4^. Efforts to identify brain regions involved in drug dependence and negative withdrawal states have implicated the ventral striatum, extended amygdala, periaqueductal gray, cingulate cortex, and median raphe nucleus^2,5,6^. However, another brain structure that is involved in processing aversion and negative emotional states, known as the lateral habenula (LHb), has more recently been implicated in some forms of withdrawal^7–10^.

Consistent with its role in processing negative affect, LHb neuronal activity increases in response to situations and stimuli associated with negative outcomes, such as the absence of expected rewards (reward prediction error, RPE)^11^, or the presence of aversive stimuli. Conversely, LHb activity decreases when unexpected rewards are received^12^. Importantly, LHb neuron activity is 180 degrees out of phase with the activity of the midbrain dopamine (DA) neurons that encode reward^10,11^, and this is mediated by LHb stimulation of inhibitory neurons targeting DA neurons^13^.

LHb output neurons are largely glutamatergic^14–16^, and evidence suggests that LHb involvement in negative affective states is mediated through its control of monoaminergic neurons, such as DA cells in the ventral midbrain and hindbrain serotonin (5-HT) cells that extensively innervate forebrain areas^7,10,17,18^. Although direct connections between the LHb and monoaminergic regions exist^14,19^, LHb-mediated inhibition of DA and 5-HT neurons is thought to occur via excitation of GABAergic rostromedial tegmentum (RMTg) neurons that target the monoaminergic cells^13,19–24^. These studies, together with those showing that LHb neurons are sensitive to opioids, cocaine, alcohol, nicotine and cannabinoids, have led to the hypothesis that changes in LHb activity may underlie some acute aversive effects of these drugs, as well as the negative affective states observed during withdrawal from long-term exposure^2,6,17,25–29^.

Although strong evidence supports the idea that drug withdrawal is associated with negative physiological and psychological states, only recently has cannabis use disorder (CUD) and the existence of a cannabis withdrawal state been codified in humans in the Diagnostic and Statistical Manual of Mental Disorders (DSM-5)^30,31^. Thus, cessation of long-term use of natural and manufactured sources of Δ^9^-THC results in behavioral withdrawal symptoms and changes in brain function similar to those observed for withdrawal of other addictive drugs. For example, diminished forebrain DA function during drug withdrawal is observed with opioids and cocaine, as well as cannabis^27,32–34^. More generally, however, the consequences of cannabis withdrawal on brain function are poorly understood compared to those of other psychoactive drugs.

Cannabinoid CB1Rs mediating the effects of Δ^9^-THC have been identified in the LHb, where they alter synaptic transmission and behavior^25,35–37^. Moreover, withdrawal from long-term Δ^9^-THC exposure has recently been shown to reduce LHb neuron spontaneous firing, supporting the idea that the LHb may be involved in the negative affective state seen during cannabis withdrawal^27^. Despite advances in our knowledge of the consequences of CUD and descriptions of cannabis withdrawal, the contribution of the LHb to this state and how its function may be altered remain unknown. Here, we demonstrate that inhibitory synaptic inputs controlling LHb neuron excitability are impaired during Δ^9^-THC withdrawal and that this contributes to heightened behavioral responses to aversive stimuli to influence fear learning.

## Results

### Increased footshock-evoked LHb neuron calcium signal during Δ^9^-THC withdrawal

**Fig. 1:**
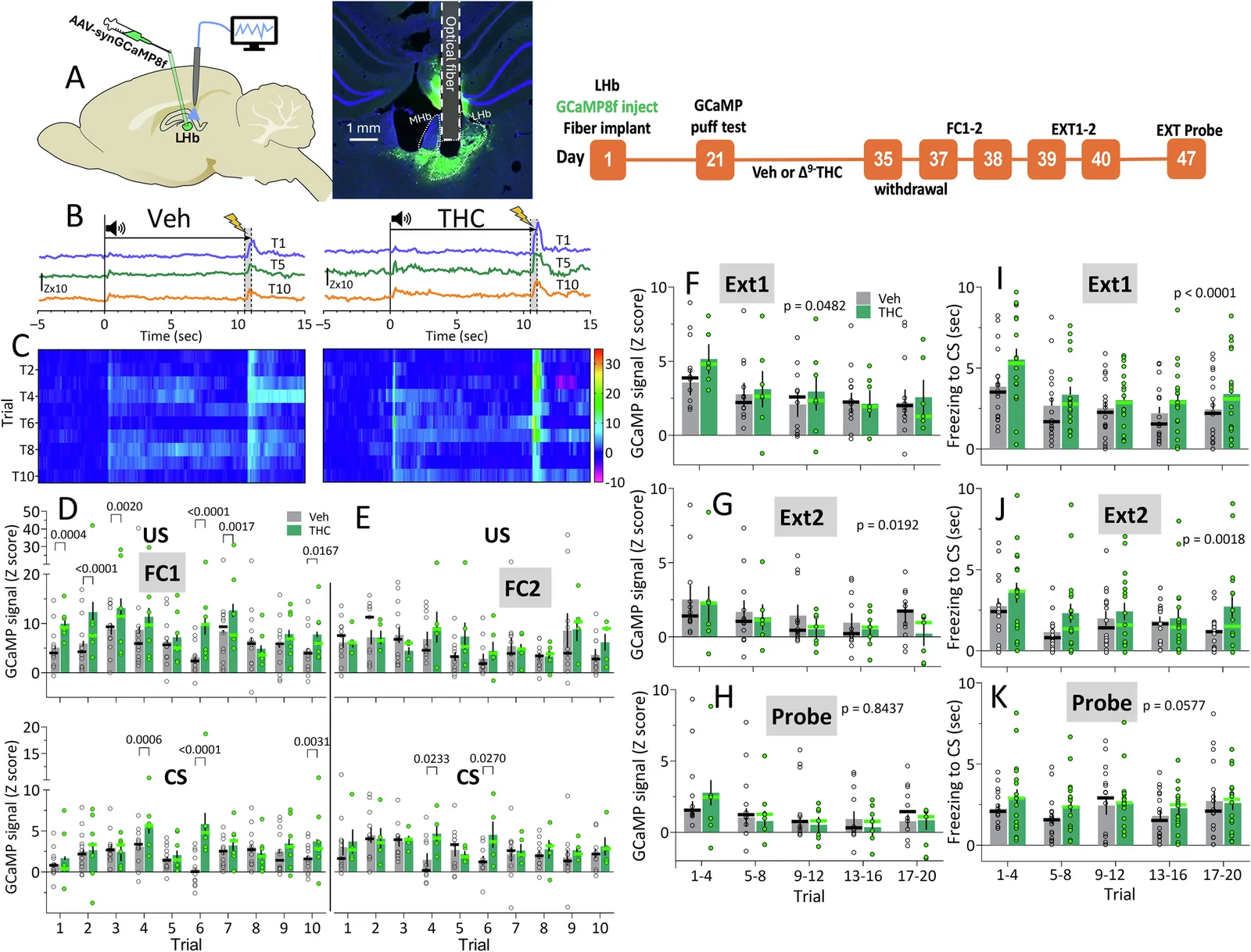
LHb GCaMP8f fluorescence during fear conditioning (FC) in Δ^9^-THC or Veh-withdrawn rats. **A** Experimental setup diagram with representative GCaMP8f expression and timeline. Sagittal brain image modified from Jimmy Dooley (CC-BY 4.0. SciDraw.io. https://scidraw.io/drawing/mid-sagittal-rat-brain-45870aff. **B** Mean GCaMP8f z-scores during trials 1, 5, and 10 of fear conditioning session 1 (FC1), 2-d after withdrawal of Veh (*n* = 10) or Δ^9^-THC (*n* = 10). Tone onset at 0 sec, footshock at 10.5-11 sec (shaded bar). **C** Same time course as **B**. FC1 heatmaps of mean GCaMP8f z-scores across trials during Veh (=12) or Δ^9^-THC (*n* = 7) withdrawal (scale: z-score). **D** Top: mean (±SEM; medians as horizontal bars) GCaMP8f z-scores during footshock (US). Significant trial x treatment (F_9,170_ = 3.38, *p* = 0.0009); differences at trials 1-3,6-7, 10 (Šídák’s); significant main effects; trial (F_9,170_ = 8.67, *p* < 0.0001), treatment (F_1,170_ = 62.76, *p* < 0.0001). Bottom: FC1, Mean GCaMP8f z-score during CS. Significant trial x treatment (F_9,170_ = 7.64, *p* < 0.0001), and treatment and trial main effects (F_1,170_ = 31.69, *p* < 0.0001; F_9,170_ = 5.388, *P* < 0.0001); differences at trials 4, 6, 10 (Šídák). **E** FC2: Top, GCaMP8f mean z-scores during US. No significant interaction (F_9,140_ = 0.536, *p* = 0.847) or main effects. Bottom: GCaMP8f responses to CS, no significant interaction (F_9,140_ = 0.854, *p* = 0.568); significant treatment effect (F_9,140_ = 4.19, *p* = 0.0426); differences at trials 4 and 6 (Šídák). **F** GCaMP8f z-score in extinction 1 (Ext1, 5 trial averages). No significant interaction (F_19,340_ = 0.6678 *p* = 0.850); significant trial (F_19,340_ = 1.917, *p* = 0.0123) and treatment main effects (F_1,340_ = 3.933, *p* = 0.048); differences at trial 2 (p = 0.0305, Šídák’s). **G** Ext2: GCaMP8f z-score. No significant interaction (F_19,330_ = 0.4533, *p* = 0.9779), no significant trial main effect (F_19,330_ = 1.416, *p* = 0.1163), significant treatment main effect (F_1,330_ = 5.537, *p* = 0.0192). **H** Probe test (Ext3) GCaMP8f z-scores: No significant interaction (F_19,300_ = 1.126, *p* = 0.3235); significant trial main effect (F_19,300_ = 2.912, *p* < 0.0001; differences on trials 1 and 2, p = 0.0087, *p* = 0.0277, Šídák). No significant treatment effect (F_1,300_ = 0.039, *p* = 0.8437). **I** Ext1: Time freezing during CS. No significant interaction (F_19,680_ = 1.074, *p* = 0.3738), significant trial (F_19,680_ = 3.950, *p* < 0.0001) and treatment (F_1,680_ = 19.36, *p* < 0.0001) main effects. Differences at trials 1-3, 5, 17 (*p* = 0.0096, 0.00263, 0.0432, 0.0088, 0.0315; Sidak’s, *n* = 19, and 17). **J** Ext2: Freezing to CS. No significant interaction (F_19,680_ = 0.5745, *p* = 0.9250), significant trial (F_19,680_ = 1.721, *p* = 0.0287) and treatment main effects (F_1,680_ = 9.794 *p* = 0.0018). Difference at trial 2, *p* = 0.0370, Šídák. **K** Probe extinction test (Ext3); no significant interaction (F_19,680_ = 0.5773, *p* = 0.9233), no significant main effects (*n* = 18, Veh, THC). **D**–**K** All data are means (±SEM), medians as horizontal bars. All F*-*values from 2-way ANOVA. All statistical tests throughout are summarized in Supplementary Data 1.

**Fig. 2:**
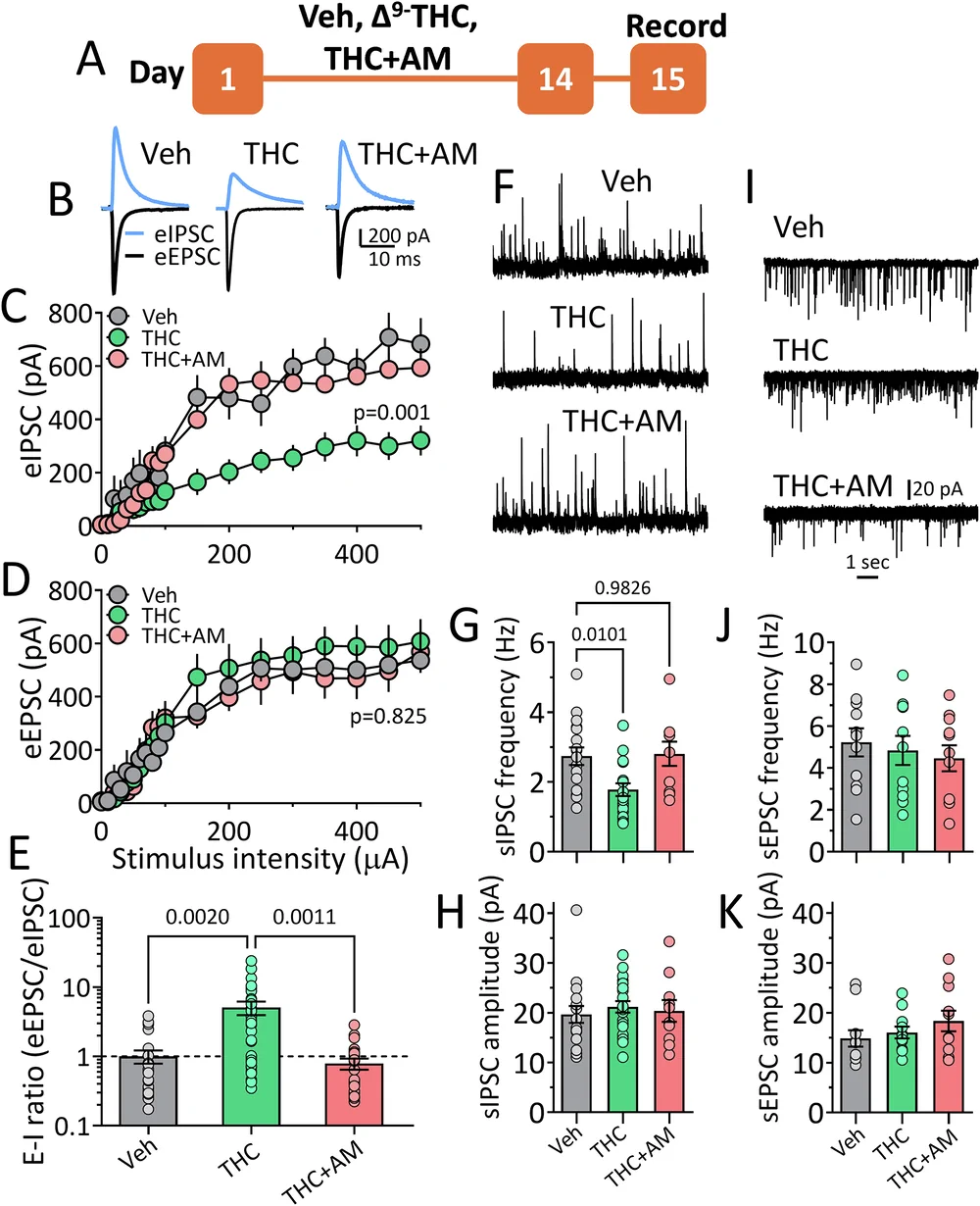
Disruption of E-I balance of synaptic input to LHb neurons during Δ^9^-THC withdrawal. **A** Experimental timeline. **B** Averaged electrically-evoked eIPSCs (blue, Vh = 0 mV) and eEPSCs (black, Vh = –70 mV) recorded simultaneously from LHb neurons in rats withdrawn from Veh, THC, or THC + AM treatments. **C** eIPSC I-O curves show significantly reduced inhibition during THC withdrawal, prevented by AM251 co-treatment (2-way ANOVA, interaction: F_36,646_ = 2.634, *p* < 0.0001; main treatment effect, F_2,646_ = 66.47, *p* < 0.0001). **D** No treatment effect on eEPSC I-O curves (2-way ANOVA, interaction: F_36,646_ = 0.3624, *p* = 0.999; main treatment effect, F_2,646_ = 1.743, *p* = 0.1759). **E** THC withdrawal increased the E-I ratio (eEPSC/eIPSC, 1-way ANOVA, F_2,76_ = 9.384, *p* = 0.0002), blocked by AM251 (log y-axis). Šídák post hoc values shown. **F** Representative sIPSC sweeps. **G** THC withdrawal decreased sIPSC frequency, prevented by AM251 (F_2,46_ = 6.254, *p* = 0.004, post hoc *p* values shown). **H** No change in sIPSC amplitude (1-way ANOVA, F_2,46_ = 0.2785, *p* = 0.7582). **I** Representative sEPSC sweeps. **J**, **K** No differences in sEPSC frequency or amplitude (1-way ANOVAs, F_2,30_ = 0.3293, *p* = 0.7220; F_2,30_ = 1.131, *p* = 0.3360, respectively). number rats/number neurons: **C**, **D**: 4/9 (Veh), 5/15 (THC), 5/13 (THC + AM); **E**: 9/23, 10/34, 9/23; **G**, **H**: 6/17, 6/22, 5/10; **J**, **K**: all groups 4/11. All error bars SEM.

To determine whether the sensitivity of LHb neurons to aversive stimuli is altered during Δ^9^-THC withdrawal, we used in vivo fiber photometry to measure GCaMP8f-mediated calcium responses during Pavlovian fear conditioning (FC). A mild footshock (US, 0.5 sec, 0.65 mA) was paired with an auditory tone (CS) and GCaMP8f calcium responses were measured beginning 2-d after drug or vehicle (Veh) withdrawal (Fig. 1A). FC (CS-US association) was conducted in 10, 20-sec trial blocks (Fig. 1B, C) on 2 consecutive days (FC1, FC2; Fig. 1A**)**. Consistent with previous reports in naïve rodents^38,39^, footshock was associated with a significant increase in LHb calcium signal in both treatment groups during FC1 (Fig. 1B–D; 2-way ANOVA, F_9,170_ = 8.67, *p* < 0.0001, main effect of trial). However, this was significantly larger in the Δ^9^-THC withdrawal group (Figs. 1B–D, 2-way ANOVA, time x treatment interaction, F_9,170_ = 3.38, *p* = 0.0009, and treatment main effect = F_1,170_ = 62.762, *p* < 0.0001), with differences seen on trials 1–3, 6–7, and 10 (*p* = 0.0004, *p* < 0.0001, *p* = 0.002, *p* < 0.0001, *p* = 0.017, *p* = 0.0167 respectively, Šídák’s post-hoc). However, the calcium response did not differ between groups in response to the US during FC2 (Fig. 1E, top; treatment x trial interaction, F_9,140_ = 0.536, *p* = 0.847, main effect of treatment, F_1,140_ = 0.5709, *p* = 0.4512). The data suggest that the excitation of LHb neurons to US presentation was greater in the Δ^9^-THC withdrawal group during the first day of fear conditioning. This statistical information and all statistical tests are summarized in Supplementary Data 1.

### Enhanced CS-evoked GCaMP8f signal in the LHb during Δ^9^-THC withdrawal

Peak GCaMP8f signals were also measured during 10 pairings of the CS tone with footshock during FC1 and FC2 in both the Veh and Δ^9^-THC withdrawal groups (Fig. 1B–E). GCaMP8f responses recorded during the first 1 sec of CS were initially small or absent but increased with additional CS-US pairings in both treatment groups (Fig. 1B–E, FC1, trial main effect, F_9,170_ = 5.388, p < 0.0001), and when collapsed across all trials during FC1 both groups showed a significant increase in GCaMP8f signal upon CS presentation (Fig. S1A, one sample t-test versus z-score = 1.0, Veh, t_9_ = 3.801, p = 0.0042, Δ^9^-THC = t_9_ = 5.181, *p* = 0.0006), indicating that the CS-US association was encoded by LHb neurons in both groups. Further analysis showed that the CS GCaMP8f signal was significantly larger in the Δ^9^-THC withdrawal group (Fig. S1B, collapsed across all trials, unpaired t-test, t_18_ = 2.206, *p* = 0.046; Fig. 1D, bottom graph, 2-way ANOVA, trial x treatment interaction, F_9, 170_ = 7.64, *p* < 0.0001), with differences on trials 4, 6, and 10 (Šídák’s, *p* = 0.0006, *p* < 0.0001, p = 0.0031, respectively). Differences in CS-evoked calcium signals between treatment groups were less apparent during FC2, with no significant trial x treatment interaction (F_9, 140_ = 0.854, *p* = 0.568), but a significant treatment effect (F_1, 140_ = 4.19, *p* = 0.043; differences on trials 4 and 6, Šídák’s, *p* = 0.0233 and *p* = 0.0270). This treatment effect was also observed when the FC2 calcium data were collapsed across trials (unpaired t-test, t_18_ = 2.156, *p* = 0.0449). The results suggest that LHb neurons encode the association between an aversive US and a neutral CS, and that this association is stronger during Δ^9^-THC withdrawal.

### Extinction of LHb CS-evoked GCaMP8f signals during Δ^9^-THC withdrawal

Extinction of the CS-US association was assessed across 2 daily sessions of 20 CS presentations (Ext1, Ext2), beginning 1 d after FC2 (Fig. 1A, F, G). The CS-associated calcium signals decreased across Ext1-2 in both withdrawal groups (Ext1, trial main effect, F_19,340_ = 1.917, *p* = 0.0123, Fig. 1F, G), and there was a significant main effect of treatment (F_1,340_ = 3.933, *p* = 0.0482), but no trial x treatment interaction during Ext1 (F_19,340_ = 0.6678, *p* = 0.8504). This effect was not observed when data were collapsed across trials (Fig. S1D, unpaired t-test, t_38_ = 1.486, *p* = 0.1457). A significant trial x treatment interaction was not observed for Ext2 GCaMP8f signals (F_19,330_ = 0.4533, *p* = 0.9779), and groups did not differ across trials (trial main effect, F_19,330_ = 1.416, *p* = 0.1163). However, the calcium signals were unexpectedly significantly smaller in the Δ^9^-THC withdrawal group during Ext2 (Fig. 1G, treatment main effect, F_1,330_ = 1.357, *p* = 0.0192; Figure S1E, unpaired t-test, collapsed across all trials, t_38_ = 2.247, *p* = 0.0305).

Seven days following Ext2, a CS probe test (Ext3) was conducted in a novel environment to measure extinction recall (Figs. 1A, 1H). The GCaMP8f signals from Δ^9^-THC withdrawal animals (12 days after drug cessation), did not differ significantly from the Veh group (trial x treatment interaction, F_19,300_ = 1.126, *p* = 0.325; treatment main effect, F_1, 300_ = 0.03892, *p* = 0.8437). The same data pooled across trials and subjects showed no significant effect of treatment (Figure S1F). These data show that CS-associated calcium signals were significantly elevated in the Δ^9^-THC withdrawal group only during the early trials of Ext1.

### Increased freezing response to CS during Δ^9^-THC withdrawal during extinction

Behavioral measurement of FC recall was conducted during extinction sessions where the CS was presented in the absence of the US (Fig. 1I–K). Although a significant interaction between withdrawal condition and trial was not observed during Ext1 (F_19,680_ = 1.074, *p* = 0.3738), there were significant main effects of treatment (F_1,680_ = 19.36, *p* < 0.0001) and trial (F_19,680_ = 3.95, *p* < 0.0001), with significance detected on trials 1, 2, 3, 5, 17 (*p* = 0.0096, 0.00263, 0.0432, 0.0088, and 0.0315, Šídák’s post-hoc). Thus, Δ^9^-THC withdrawal rats exhibited more freezing compared to Veh controls during Ext1 (Fig. 1I). A significant interaction between treatment group and trial was not observed during Ext2 (F_19,680_ = 0.5745, p = 0.9520, Fig. 1J). However, significant main effects of treatment and trial were observed (F_19,680_ = 9.794, *p* = 0.0018; F_19,680_ = 1.721, *p* = 0.0287, respectively; Fig. 1J), with differences detected on trial 7 (*p* = 0.0370, Šídák; Fig. 1J). These data indicate greater freezing times during Ext2 by rats in the Δ^9^-THC withdrawal group. To further assess extinction, a recall probe test (Ext3) was performed 1 week following Ext2 in a novel environment in which the grid floor of the operant chamber was covered with smooth opaque Plexiglas. Although neither significant trial x treatment interaction (F_19,680_ = 0.5733, *p* = 0.925), nor significant main effects were observed (F_19,680_ = 0.7185, *p* = 0.8017; F_19,680_ = 3.616, *p* = 0.0577, trial or treatment main effects, respectively), there was a trend toward increased freezing in the Δ^9^-THC withdrawal group during trial blocks 1–4, and 5–8 (Fig. 1K). Extinction freezing times were next collapsed across trials within session and analyzed by 2-way ANOVA (Fig. S2). This showed that the Δ^9^-THC withdrawal group exhibited significantly more freezing during Ext1-2 (2-way ANOVA, treatment main effect F_1, 2154_ = 37.92, p < 0.0001), and that mean freezing times decreased in both treatment groups across all extinction sessions (extinction session main effect = F_2, 2154_ = 26.32, *p* < 0.0001, Fig. S2). Together, these data show that Δ^9^-THC withdrawn rats exhibited more freezing during extinction, and that both groups showed between-session reductions in freezing behavior.

### Absence of sex differences in freezing response during Δ^9^-THC withdrawal

Freezing data were also analyzed to determine whether FC extinction during Δ^9^-THC withdrawal differed between male and female rats (Fig. S3). No significant differences were observed between males and females in either the Veh or the Δ^9^-THC withdrawal groups, whether analyzed by 2-way RM-ANOVA (main effect of sex, Veh, F_1,16_ = 0.3518, *p* = 0.5614; Δ^9^-THC, F_1,16_ = 0.8905, *p* = 0.3594, Fig. S3A, S3C), or when collapsed across all 20 extinction trials and analyzed by unpaired t-test (male vs. female, Veh, t_16_ = 0.5915, *p* = 0.5625; Δ^9^-THC, t_16_ = 0.9543, *p* = 0.3541; Fig. S3B, S3D).

### Increased freezing during Δ^9^-THC withdrawal does not result from increased sensitivity to conditioning stimuli

Our data show that LHb calcium signals were significantly larger in the Δ^9^-THC withdrawal group during FC1-2 and Ext1-2 sessions. This could reflect a general increase in reactivity to novel sensory stimuli (e.g., the CS) that could be interpreted as a heightened emotional response to the CS. Therefore, we conducted an experiment in which the CS and US were presented but were not temporally paired, and assessed freezing behavior during CS presentation during extinction. Two 10-trial sessions were conducted on 2 consecutive days in which footshocks were delivered at random intervals (20–90 sec) at the end of each 10 sec CS presentation. The extent of CS-associated freezing was then assessed during 3 sessions of 20 trials of CS presentations (Fig. S4). There were no significant differences between Veh and Δ^9^-THC withdrawal groups during the 3-extinction session (Fig. S4 shows results from 2-way ANOVAs, treatment main effect across Ext1-3). This supports the idea that the significantly longer freezing times in the Δ^9^-THC withdrawal group (Fig. 1) did not result from a heightened sensory response to the CS.

### Increased LHb neuron excitability and impaired inhibitory input during Δ^9^-THC withdrawal

To determine a potential mechanism for the increased response of LHb neurons to aversive stimuli during Δ^9^-THC withdrawal, we performed synaptic analyses using whole-cell electrophysiology in brain slices. Three groups of rats were used; each received 14 daily i.p. injections of either Veh, Δ^9^-THC (5 mg/kg) alone, or the CB1R antagonist AM251 (1 mg/kg), followed 30 min later by injection of 5 mg/kg Δ^9^-THC (Veh, THC, THC + AM groups). Twenty-four hours after the final injection, cellular and synaptic properties of LHb neurons were assessed.

Glutamatergic EPSCs and GABAergic IPSCs were recorded from LHb neurons in vitro using whole -cell patch clamp. Electrically-evoked IPSCs (eIPSCs) were recorded at a holding potential (Vh) of 0 mV, and electrically-evoked EPSCs (eEPSCs) at Vh = −70 mV, in the same LHb neurons (Fig. 2B). Input-output (I-O) curves plotting the relationship between stimulus intensity and synaptic response amplitude showed that eIPSCs in neurons from the Δ^9^-THC withdrawal group were significantly smaller across a range of stimulus intensities, compared to Veh and AM251 + THC groups (Fig. 2C, 2-way ANOVA, stimulus intensity x treatment interaction, F_36,646_ = 2.634, *p* < 0.0001, treatment main effect, F_2,646_ = 66.47, *p* < 0.0001). In contrast, glutamatergic eEPSCs did not differ among the 3 treatment conditions (Figs. 2D, 2-way ANOVA, stimulus x treatment interaction = F_36,646_ = 0.3624, *p* = 0.999; main effect of treatment, F_2,646_ = 1.743, *p* = 0.1759). To determine whether the deficit in LHb inhibitory transmission during Δ^9^-THC withdrawal resulted from diminished GABA release probability, spontaneously occurring IPSCs (sIPSCs) were measured in LHb neurons from each of the treatment groups. The frequency of sIPSCs was significantly lower in LHb neurons from Δ^9^-THC withdrawal rats, and this was prevented by AM251 co-administration with the phytocannabinoid in vivo (Fig. 2F, G, 1-way ANOVA, F_2,46_ = 6.254, *p* = 0.0040, Šídák’s post-hoc, Δ^9^-THC vs. Veh, *p* = 0.0101, Veh vs. THC + AM, *p* = 0.983). In contrast, sIPSC amplitudes did not differ among the 3 treatment conditions (Figs. 2H, 1-way ANOVA, F_2,46_ = 0.28, *p* = 0.758). Spontaneous EPSCs from LHb neurons obtained from rats in each of the 3 treatment groups were also measured, and neither their frequency nor amplitude differed significantly (Figs. 2I–K, 1-way ANOVA, sEPSC frequency, F_2,30_ = 0.3293, *p* = 0.722; sEPSC amplitude, F_2,30_ = 1.13, *p* = 0.336). Another useful measure of altered synaptic integration that has translational value due to its use in establishing baseline levels excitability and their contribution to changes in circuit bias^40^ is known as the excitation/inhibition ratio (E-I). We calculated E-I ratios from eIPSCs and eEPSCs recorded in the same neurons and observed roughly similar levels of excitation and inhibition in the Veh control group (E-I ratio = 1.0 ± 0.2, *n* = 23 cells, Fig. 2E). However, this ratio was significantly larger in neurons from in Δ^9^-THC withdrawal rats (E-I ratio = 5.05 ± 1.12, *n* = 33 cells; 1-way ANOVA, F_2,76_ = 9.38, *p* = 0.0002, Fig. 2E), and this was prevented by the AM251 injections preceding Δ^9^-THC (E-I ratio = 0.787, ± 1.14, *n* = 23 cells, *p* = 0.984, Šídák’s post hoc test, Fig. 2E). Therefore, these data show that impaired GABA release in the LHb during Δ^9^-THC withdrawal was associated with a large increase in the E-I ratio and thus increased LHb excitability.

### Basal forebrain inhibition of the LHb is selectively impaired during Δ^9^-THC withdrawal

**Fig. 3:**
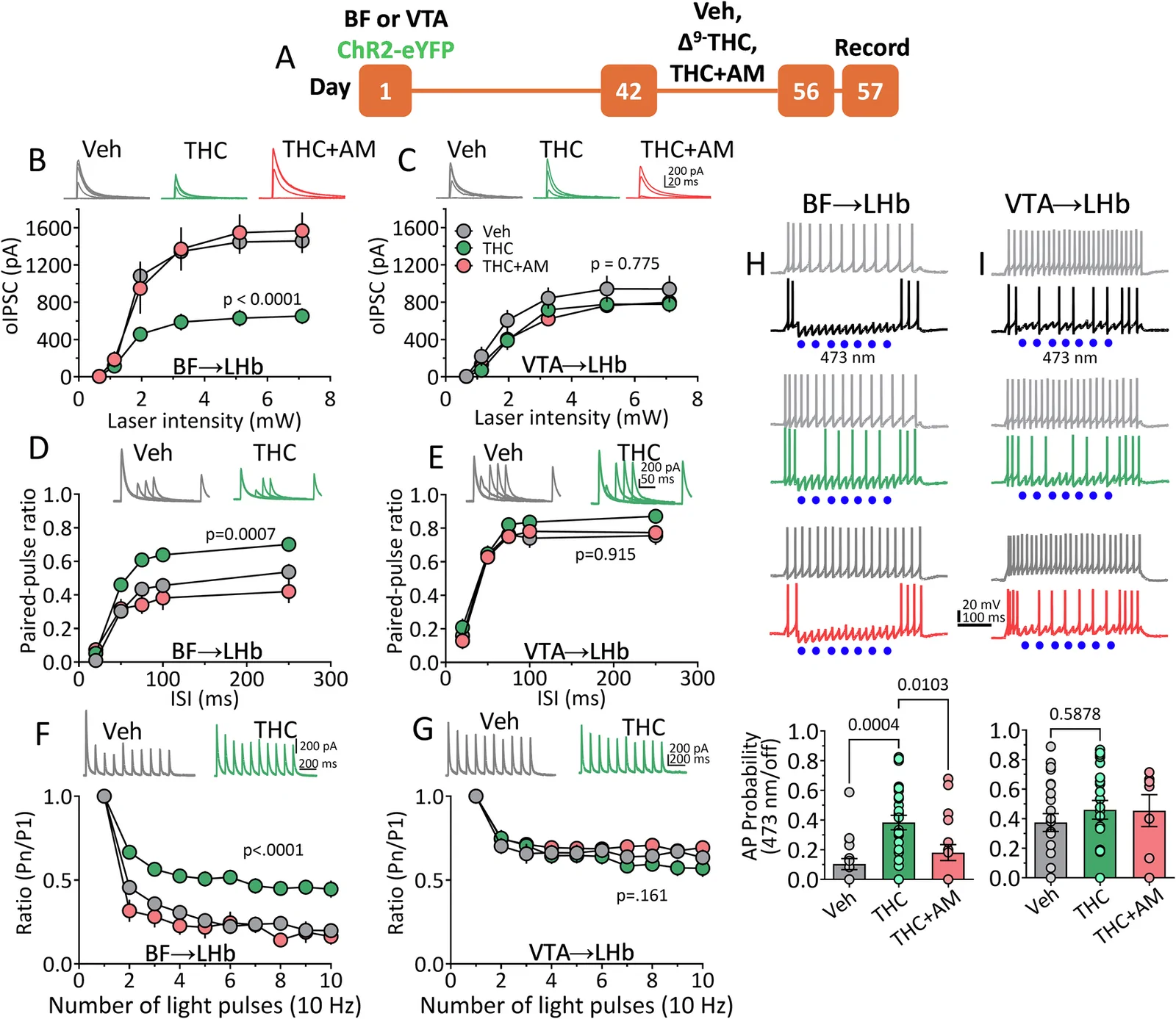
Δ⁹-THC withdrawal alters BF– but not VTA–driven synaptic inhibition of LHb neurons. **A** Experimental timeline. I-O curves and averaged oIPSCs from BF (**B**) and VTA (**C**) inputs show reduced BF oIPSCs during Δ⁹-THC withdrawal and no change at VTA inputs (**B**, 2-way ANOVA, interaction, F_(10, 192)_ = 4.730, *p* < 0.0001; treatment main effect, F_2,192_ = 45.62, *p* < 0.0001; **C** interaction, F_4.470, 60.34_ = 0.6427, *p* = 0.6510; treatment main effect, F_2, 27_ = 1.299, p = 0.2893). **D**, **E** Paired-pulse ratios (PPRs) across interstimulus intervals (ISI). PPR was increased at BF inputs after THC withdrawal (2-way ANOVA, interaction, F_8,190_ = 2.151, *p* = 0.0330; treatment main effect, F_2, 190_ = 30.57, *p* < 0.0001), indicating decreased release probability; PPR was unchanged at VTA inputs (main effect of treatment, F_2, 29_ = 1.512, *p* = 0.2373). AM251 co-treatment normalized BF PPR. 10 Hz photostimulation revealed reduced short-term synaptic depression at BF inputs during THC withdrawal (**F**, 2-way ANOVA, interaction, F_18,288_ = 5.699, *p* < 0.0001, treatment main effect, F_2,32_ = 13.97, p < 0.0001), but not at VTA inputs (**G**, treatment main effect, F_2, 26_ = 0.6879, *p* = 0.5115). BF inputs showed greater depression than VTA under control conditions (unpaired t-test, t = 2.266, df=18, *p* = 0.036), suggesting higher release probability. Current clamp recordings of action potentials (APs) in LHb neurons during current injection with/without photostimulation of BF (**H**) or VTA (**I**) inputs. THC withdrawal reduced BF-mediated AP suppression (1-way ANOVA, F_2, 61_ = 9.485, *p* = 0.0003), prevented by AM251 (*p* = 0.0112, Šídák post hoc); no effect on VTA-driven AP suppression (F_2,51_ = 0.5390, p = 0.5866). number rats/number neurons: B: 5/12 (Veh), 5/15 (THC), 4/8 (THC + AM); C: 4/9, 5/12, 5/9; D: 6/17, 6/17, 4/7; E: 6/9, 6/12, 5/11; F: 6/11, 7/15, 4/7; G: 6/9, 6/11, 5/9; H: 6/18, 8/27, 6/19; I: 7/24, 7/23, 4/7. **B**–**I**. Data are means (±SEM).

Inhibitory control of LHb neurons is largely derived from extrinsic afferents^41^, and we recently identified a GABAergic projection originating in the rostral ventral pallidum (VP) and caudal nucleus accumbens shell (NAcs) of the basal forebrain (BF) that provides strong inhibition of LHb neurons and is controlled by CB1Rs^25^. Previous studies also show that distinct afferents to LHb neurons are not uniformly sensitive to CB1R activation^25,35^, and that inhibition of LHb neurons by ventral tegmental area (VTA) afferents is not altered by cannabinoids^25^. Therefore, to determine whether GABAergic BF inputs to the LHb are impaired during Δ^9^-THC withdrawal, and whether the impairment requires CB1R expression, we examined the integrity of BF and VTA inhibitory inputs to the LHb during Δ^9^-THC withdrawal. Separate groups of rats received injections of a channelrhodopsin-2-expressing virus (ChR2, AAV5-hSyn-ChR2-eYFP) into either the BF (Fig. S6) or the VTA and approximately 6 weeks later underwent long-term treatment with Veh, Δ^9^-THC, or AM251 + Δ^9^-THC. Beginning at 24 hours of withdrawal from treatment, photostimulated optical IPSCs (oIPSCs) were measured in LHb neurons in vitro. Input-output relationships for oIPSCs evoked from BF inputs showed that these synaptic currents were significantly smaller in LHb neurons in the Δ^9^-THC withdrawal group, compared to the Veh, or THC + AM groups (Fig. 3B, 2-way ANOVA, treatment x light (mW) interaction, F_10,192_ = 4.73, *p* < 0.0001; main effect of treatment, F_2,192_ = 45.62, *p* < 0.0001; Šídák’s post hoc, THC vs. Veh and THC vs. THC + AM251, *p* < 0.0001). In contrast, inhibitory inputs to LHb from VTA were unaltered in the Δ^9^-THC and THC + AM withdrawal groups, compared to Veh controls (Fig. 3C, 2-way ANOVA, treatment x light (mW) interaction, F_4,60_ = 0.643, *p* = 0.6510; treatment main effect, F_2,27_ = 1.299, *p* = 0.2893). To determine whether the altered I-O relationship at BF to LHb inputs occurred via a presynaptic mechanism, paired oIPSC responses, evoked by 2 rapid photostimulations of ChR2 at several interstimulus intervals (ISI) were measured at both BF and VTA projections. The ratio of the first and second oIPSC amplitudes was then calculated as an index of presynaptic function (paired-pulse ratio, PPR = oIPSC2/oIPSC1, Fig. 3D). Mean PPRs evoked by BF afferent stimulation were less than unity in Veh withdrawal neurons, indicating paired-pulse inhibition, and were significantly larger in the Δ^9^-THC withdrawal group (Figs. 3D, 2-way ANOVA, ISI x treatment interaction, F_8,190_ = 2.151, *p* = 0.0330; main treatment effect, F_2,190_ = 30.57 *p* < 0.0001), indicating a decrease in GABA release probability from these axons during Δ^9^-THC withdrawal. AM251 injections prevented the change in PPRs (Fig. 3D, Šídák, THC vs. THC + AM, *p* < 0.0001). In contrast to the changes observed at BF inputs to LHb, PPRs were unaltered at VTA GABAergic inputs to LHb during Δ^9^-THC withdrawal (Fig. 3E, 2-way ANOVA, ISI x treatment interaction, F_4,60_ = 0.4070, *p* = 0.8098; treatment main effect, F_2,29_ = 1.512. *p* = 0.2373).

Differences in release probability are also observed at central synapses during sustained activation of afferents, and this may be relevant to information processing during neuronal oscillations^42^. Therefore, we examined synaptic properties of VTA and BF inputs to LHb neurons during 10 Hz photostimulation and expressed the probability of GABA release as a ratio of oIPSC amplitudes evoked by light pulses 2-10 to those evoked by the first pulse (oIPSC_n_/oIPSC_1_, or P_n_/P_1_ ratio, Fig. 3F, G). A comparison between BF and VTA inputs to LHb inputs showed that the probability of GABA release was higher at BF synapses in neurons from Veh rats (i.e., stronger depression of oIPSCs, compared to VTA inputs; Fig. 3F, G, unpaired t-test, t_18_ = 2.266, *p* = 0.036). Moreover, a significant increase in the Pn/P1 ratio was observed at BF-LHb synapses in Δ^9^-THC withdrawn rats (Figs. 3F, 2-way ANOVA, treatment x pulse number interaction, F_18,288_ = 5.70, *p* < 0.0001; treatment main effect, F_2,32_ = 13.97, *p* < 0.0001), compared to no change in this measure at VTA-LHb synapses (Figs. 3G, 2-way ANOVA, treatment x pulse number interaction, F_18,234_ = 1.35, *p* = 0.161; treatment main effect, F_2,26_ = 0.6879, *p* = 0.5115). Together, these data indicate that GABA release probability was significantly reduced at BF inputs, but not at VTA inputs to LHb neurons during Δ^9^-THC withdrawal.

To determine the consequence of reduced inhibitory control during Δ^9^-THC withdrawal on LHb neuron excitability, we examined effects of photostimulation of BF or VTA LHb afferents on the probability of action potential (AP) discharge in current clamp experiments (Fig. 3H, I). APs were evoked by 1 sec, 200 pA, depolarizing currents injected through patch-clamp electrodes, either in the absence or presence of 473 nm light pulse photostimulation of BF or VTA afferents in each of the treatment groups (Fig. 3H, I). Photostimulation of either BF or VTA LHb GABAergic afferents significantly reduced AP discharge probability in cells from Veh withdrawal animals (Fig. 3H, I, one sample t-test, BF input, t_17_ = 24.16, *p* < 0.0001; VTA input, t_23_ = 10.39, *p* < 0.0001). However, this suppression of AP discharge probability was significantly smaller at only the BF inputs to LHb neurons in the Δ^9^-THC withdrawal subjects, and this was prevented by AM251 pretreatment in vivo (Fig. 3H, I, BF afferents, 1-way ANOVA, F_2,61_ = 9.485, *p* = 0.0003, Veh vs. Δ^9^-THC, *p* = 0.0004, THC vs. THC + AM, *p* = 0.0112, Šídák’s post-hoc; VTA afferents 1-way ANOVA, F_2,51_ = 0.5390, *p* = 0.587). These results indicate that Δ^9^-THC withdrawal is associated with impaired GABA release at BF inputs to the LHb, that this requires CB1Rs, and that inhibitory VTA input to the LHb is unaltered, whereas the increase in AP output indicates increased LHb neuron excitability.

### Time course of the impairment of BF inhibition of the LHb during Δ^9^-THC withdrawal

**Fig. 4:**
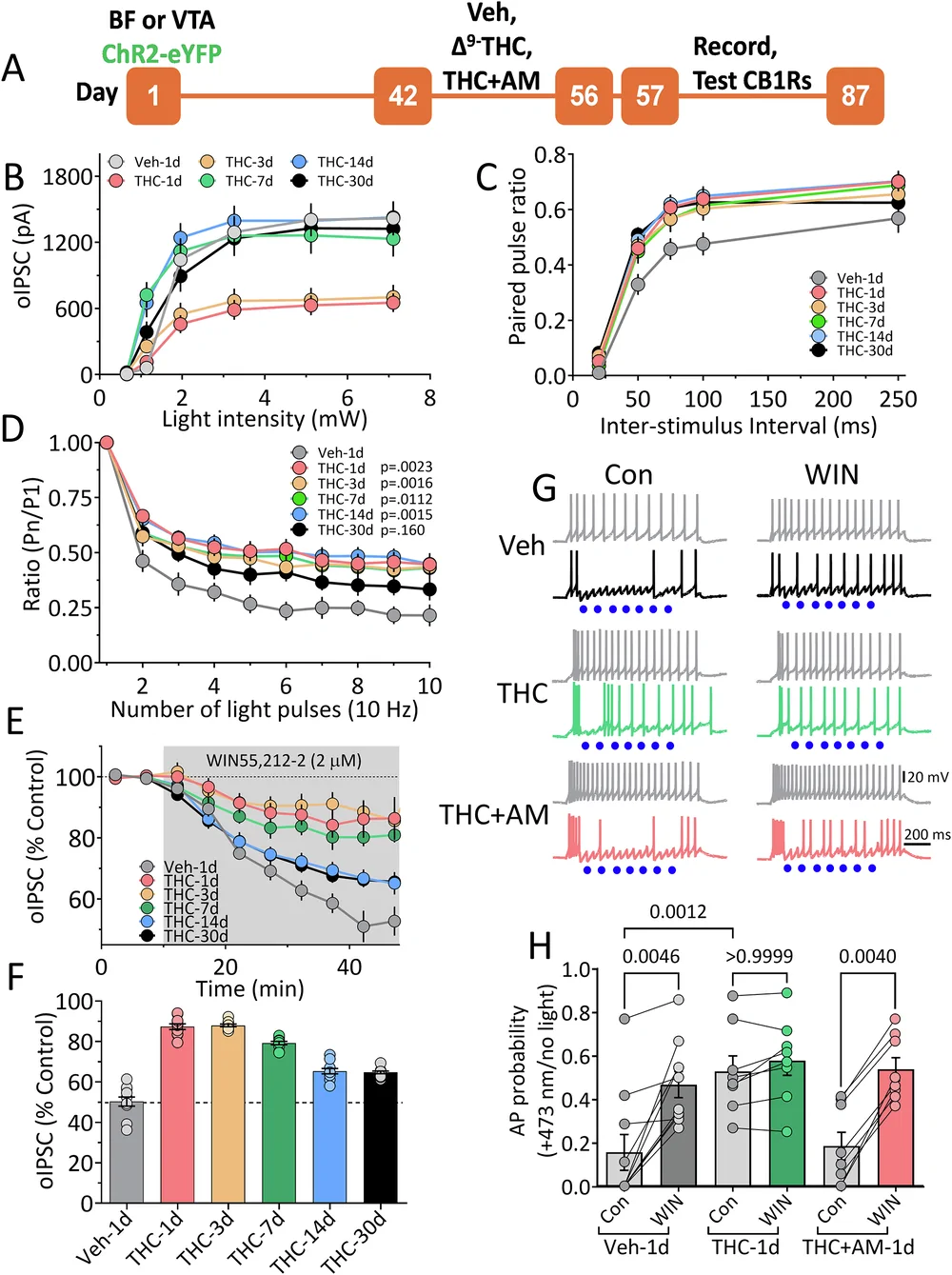
Time course of impaired BF–LHb GABAergic transmission and CB1R desensitization during Δ⁹-THC withdrawal. **A** Experimental timeline. **B** I-O curves of photostimulated BF oIPSCs at different days of Δ^9^-THC or Veh withdrawal show reduced inhibition at 1- and 3-days post-withdrawal (2-way ANOVA, interaction, F_25,468_ = 2.222, *p* = 0.0007, withdrawal day main effect, F_5,468_ = 29.16, *p* < 0.0001; Veh-1d vs. THC-1d or THC-3d, *p* < 0.0001, Šídák’s post hoc). **C** PPRs significantly increased during THC withdrawal (2-way ANOVA, main effect of withdrawal day, F_5, 425_ = 9.753, *p* < 0.0001_)_. Differences at 1d-30d of Δ^9^-THC withdrawal (vs. Veh-1d withdrawal, all *p* < 0.0001, Šídák posthoc) compared to Veh-1d showing enduring synaptic impairment. **D** 10 Hz photostimulation-induced synaptic depression was reduced at all withdrawal time points except 30 d (2-way ANOVA, withdrawal day main effect, F_5,790_ = 28.57, *p* < 0.0001). Differences at 1d-30d withdrawal vs 1 d Veh, all p < 0.0001, Šídák post hoc). oIPSCs evoked by pulses 2-10 are expressed as a proportion of oIPSC evoked by the first stimulus. **E**, **F** CB1R agonist (WIN, 2 µM) inhibition of BF oIPSCs was diminished throughout 1–30 d of Δ⁹-THC withdrawal (mixed ANOVA, F_2.241,291.4_ = 145.0, *p* < 0.0001; differences at 1d-30d withdrawal vs. 1 d Veh withdrawal, all *p* < 0.0001; **F**, 1-way ANOVA, F_5,60_ = 129.2, *p* < 0.0001; differences from Veh-1d withdrawal, *= *p* < 0.0001, Šídák posthoc). **G** Current clamp recordings show reduced BF-mediated inhibition of LHb APs at 1 d Δ^9^-THC withdrawal, and loss of WIN effect; AM251 co-treatment restored both. Waveforms show APs evoked by 200 pA, depolarizing current pulses in the absence of BF axon photostimulation (gray waveforms), and during photostimulation (blue dots), in neurons from the Veh-1d (black waveforms), Δ^9^-THC-1d (green waveforms) and THC + AM-1d (red waveforms) groups, before (Control, Con) and during WIN (2 µM) application. **H** Summary of AP probability showing a significant basal increase in the Δ^9^-THC-Con withdrawal group, compared to Veh-Con group (*p* = 0.0012, Šídák post-hoc), and the loss of the WIN-induced increase in AP probability in the Δ^9^-THC withdrawal neurons. Note that the change in AP probability was prevented by in vivo treatment with AM251. *p* values from Šídák post-hoc comparisons following a significant (1-way ANOVA, F_5,46_ = 7.682, *p* < 0.0001). All mean values ± SEM. number rats/number neurons: **B**–**D** Veh-1d: 5/10–15, THC-1d: 4/14–17, THC-3d: 4/14–15, THC-7d: 4/15, THC-14d: 4/15, THC-30d: 4/14–15; **E**, **F**: Veh-1d: 4/9, THC-1d: 4/12, THC-3d: 4/10, THC-7d: 4/11, THC-14d: 4/12, THC-30d: 4/11; **H**: Veh: 6/18, THC: 8/27, THC + AM: 6/19.

To determine the duration of impaired GABA release at BF inputs to LHb, we measured oIPSC I-O relationships at 1, 3, 7, 14, or 30 days of Δ^9^-THC withdrawal, and compared these I-O curves to those at 1-day of Veh withdrawal (Fig. 4B). The I-O curves measured at these withdrawal points were significantly different (Figs. 4B, 2-way ANOVA, light intensity vs. withdrawal day interaction, F_25,468_ = 2.222, p = 0.0007; withdrawal day main effect, F_5,468_ = 29.16, p < 0.0001), and post-hoc analysis indicated that I-O curves significantly differed from the Veh-1d group at 1- and 3-days of Δ^9^-THC withdrawal (vs. 1-day Veh, p < 0.0001).

In contrast to the I-O curves of oIPSCs evoked by single light pulses (Fig. 4B), the significant increase in PPRs seen in the 1-day Δ^9^-THC withdrawal group (Fig. 3D) did not completely reverse at 30 days of Δ^9^-THC withdrawal (2-way ANOVA, ISI x treatment interaction, F_20,425_ = 0.5386, *p* = 0.9497; withdrawal day main effect, F_5,425_ = 9.75, *p* < 0.0001; ISI main effect, F_4,425_ = 281.5, *p* < 0.0001), with significance observed at 50, 75 and 100 ms ISIs (Fig. 4C, Šídák). Post hoc tests showed significantly increased PPRs at all Δ^9^-THC withdrawal days compared to 1-day Veh (Šídák, *p* < 0.0001, all days). This GABA release deficit was also observed during sustained 10 Hz activation of BF inputs (Fig. 4D). Whereas a significant interaction between the number of pulses and the number of days of Δ^9^-THC withdrawal was not observed (2-way ANOVA, F_45,790_ = 0.5039, *p* = 0.9974), there was a significant main effect of withdrawal day (F_5,790_ = 28.57, *p* < 0.0001). Moreover, this reduction in the probability of GABA release during 10 Hz photostimulation was significantly smaller compared to 1 d Veh withdrawal neurons at all withdrawal time points (*p* < 0.0001, Šídák, Fig. 4D). The results indicate that GABA release probability is diminished at BF inputs to the LHb for at least 30 days after Δ^9^-THC withdrawal, and this impairment is more evident during higher frequency activation of BF-LHb synapses.

### Time course of CB1R desensitization at BF input to LHb during Δ^9^-THC withdrawal

Our data indicate that GABA release is impaired at BF inputs to LHb during Δ^9^-THC withdrawal, and this is prevented by co-administration of Δ^9^-THC and AM251 in vivo (Fig. 3), demonstrating that CB1Rs are necessary for the impairment. Therefore, we examined CB1R function at BF afferents across several time points of Δ^9^-THC withdrawal by measuring the inhibition of oIPSCs by the CB1R agonist WIN 55,212-2 (WIN, 2 µM) and compared this to the inhibition of oIPSCs by WIN in LHb neurons 1-day after Veh treatment (Fig. 4E, F). Consistent with results in drug-naïve rats^25^, WIN inhibited BF-evoked oIPSCs by 50.29 ± 2.3% (±SEM, *n* = 11 neurons) in the Veh-1d withdrawal group (Fig. 4E, F). However, in comparison, the response to WIN was significantly smaller in neurons from Δ^9^-THC rats at all withdrawal time points (Figs. 4F, 1-way ANOVA, F_5,60_ = 129.2, *p* < 0.0001; Šídák; significant differences at all withdrawal time points, ranging from 1 d to 30 d, *P* < 0.0001). These data indicate that CB1R inhibition of GABA release from BF-LHb afferents is impaired for at least 30 days following Δ^9^-THC withdrawal.

### Impaired CB1R control of LHb neuron excitability during Δ^9^-THC withdrawal

To determine how Δ^9^-THC-induced CB1R desensitization alters cannabinoid control of LHb neuron excitability, we measured WIN’s effects on AP firing probability during photostimulation of BF afferents (Fig. 4G, H). Similar to that shown in Fig. 3F, BF control of AP firing was measured by depolarizing LHb neurons via current injection during the presence and absence of photostimulation of ChR2-transfected BF afferents (Fig. 4G), and the change in AP probability caused by photostimulation was calculated before, and during application of WIN (2 µM) in cells from Δ^9^-THC and Veh 1-d withdrawal groups. Like the previous experiment (Fig. 3F), photoactivation of BF inputs to LHb neurons at 1-d of Veh withdrawal reduced LHb neuron AP probability, and the probability of firing was significantly increased during WIN application, indicating that CB1R inhibition of GABA release diminished the effect of photostimulation (Fig. 4G, H, 1-way ANOVA, F_5,46_ = 7.682, *p* < 0.0001, *p* = 0.0046, Šídák). In contrast, in neurons from the THC-1d withdrawal group, photoactivation of BF inputs reduced AP probability to a significantly smaller degree than that observed in the Veh-1d group (Fig. 4H, *p* = 0.0012, compared to the control Veh-1d group, Šídák post-hoc). AM251-co-treatment during in vivo Δ^9^-THC injections prevented the loss of CB1R modulation of AP probability (Fig. 4H, *p* = 0.004, 1d-THC + AM withdrawal AP probability vs AP probability during WIN application in the same group, Šídák). These results show that presynaptic CB1Rs can inhibit BF GABA release to modulate the excitability of LHb neurons, and that this inhibitory control is absent during Δ^9^-THC withdrawal.

### Unaltered passive and active LHb neuron membrane properties during Δ^9^-THC withdrawal

Potential changes in the intrinsic excitability of LHb neurons during Δ^9^-THC withdrawal were measured by examining resting membrane potentials (RMP), the relationship between membrane depolarization and AP discharge, and spontaneous AP firing pattern (using the classification system for LHb neurons by the R.W. Veh group^43,44^), in current clamp experiments (Figure S5). There was no effect of treatment on depolarization-AP relationships (Figure S5A, treatment main effect, 2-way ANOVA, F_2, 72_ = 1.096, *p* = 0.3399). Similarly, LHb neuron RMPs, measured immediately after patch rupture, did not differ among treatment groups (Figs. S5B, 1-way ANOVA, F_2,140_ = 0.2903, *p* = 0.7485, Šídák). LHb neurons exhibit several distinct patterns of spontaneous action potential discharge^44^, and we examined potential changes in these patterns at 1 d of Δ^9^-THC withdrawal (Fig. 1C). Although fewer silent cells (no spontaneous APs immediately after whole-cell access) and more bursting cells were observed in LHb after Δ^9^-THC withdrawal, overall, there was no significant effect of treatment on the pattern of spontaneous cell firing among the treatment groups (Fig. S5C, Chi-square value = 8.193, *p* = 0.2243). These data suggest that Δ^9^-THC withdrawal did not alter passive or active LHb neuron membrane properties under these recording conditions.

## Discussion

Withdrawal from long-term use of many addictive drugs is associated with negative emotional states that include dysphoria, increased anxiety, elevated responding to aversive stimuli, irritability, and an increased stress response^1^. Moreover, avoiding withdrawal symptoms through continued drug use impedes abstinence^1^. As the LHb encodes aversive states^9^, can strongly inhibit DA neurons when aversive stimuli are present or when expected rewards are not obtained^8^, and because CB1Rs are found within LHb circuits^25,36,45^, we hypothesized that this structure might contribute to cannabis withdrawal-associated negative affect, similar to that described in humans^46^. To evaluate this, we measured calcium responses with GCaMP8f to track LHb neuron excitability during FC in rats experiencing withdrawal from Δ^9^-THC. We found that LHb neuron GCaMP8f signals were significantly larger in Δ^9^-THC withdrawal rats during presentation of an aversive primary US and during CS presentation after CS-US pairings. The data suggest that LHb neuronal excitability increases in response to aversive events during Δ^9^-THC withdrawal, and this increased neuronal activity facilitates encoding of a previously neutral environmental stimulus associated with the US. Freezing behavior was also increased in Δ^9^-THC-withdrawn rats during extinction sessions 1-2, which is consistent with a recent report in rats experiencing Δ^9^-THC withdrawal after cannabis vapor exposure^47^.

Previous studies show that deletion or blockade of CB1Rs impairs fear conditioning extinction without affecting acquisition or consolidation of this memory^48^. In our study, increased freezing times observed in Δ^9^-THC withdrawn rats might reflect the slowing of the extinction time course, a stronger initial fear memory, increased emotional reactivity to fearful stimuli, or a general increase in sensitivity to novel stimuli. Our unpaired CS-US experiment demonstrated that increased freezing during Δ^9^-THC withdrawal does not appear to result from hypersensitivity to novel stimuli. Also, in contrast to that observed with CB1R knockout mice^48^, we found that freezing responses during Ext1-2 were larger in the Δ^9^-THC withdrawal group, suggesting that the Δ^9^-THC withdrawn animals retain a stronger initial memory of the fearful stimulus, rather than delayed extinction. This is further supported by the lack of a 2-way interaction between Veh and Δ^9^-THC withdrawal groups across the 3 extinction sessions, indicating that extinction proceeded at a similar rate in these groups (Fig. S2). Therefore, the enhanced response of LHb neurons to the US and CS and the increased extinction freezing response support the idea that Δ^9^-THC withdrawal increases emotional reactivity to primary aversive stimuli, thereby promoting a stronger learned fear response to conditioned stimuli.

To seek potential mechanisms for the increased excitability of LHb neurons to aversive stimuli during Δ^9^-THC withdrawal, we conducted in vitro electrophysiological experiments. Consistent with increased LHb GCaMP8f signals in vivo, E-I ratios were significantly elevated during Δ^9^-THC withdrawal, indicating greater excitability of LHb neurons. This shift in E-I balance resulted from impaired synaptic inhibition of LHb neurons, with no change in excitatory input, and was prevented by AM251 given prior to each Δ^9^-THC injection, indicating that impairment of inhibitory control of LHb neurons by Δ^9^-THC requires CB1Rs. Similar to our findings, a shift in LHb E-I balance toward greater excitation and impaired GABA release has been observed following mild traumatic brain injury^49^, and increased LHb E-I balance is associated with reductions in grooming, increased depressive and anxiety behaviors, suggesting that this synaptic dysregulation contributes to motivational and affective deficits^7,49^.

Prior studies from our lab and others show that LHb neuron afferents are not uniformly sensitive to CB1R activation. Of those tested, GABAergic inputs from BF (VP, NAcs, horizontal diagonal band) and lateral preoptic area (LPO) are inhibited, whereas VTA and entopeduncular nucleus (EPN) afferents are insensitive to cannabinoids^25,35^. Here, using optogenetics, we examined whether GABAergic BF or VTA LHb inputs were altered during Δ^9^-THC withdrawal. We found that GABA release probability was reduced at BF-LHb synapses, whereas release at the VTA-LHb input was unaffected. A functional consequence of this synaptic impairment was the reduced control of LHb neuron firing by photostimulated BF afferents. Collectively, the results suggest that the GABA release deficit occurs only at axons expressing CB1Rs and that BF inhibitory control of LHb excitatory output is impaired during Δ^9^-THC withdrawal, resulting in disinhibition of LHb neurons.

An impairment in GABA release has also been observed at EPN inputs to LHb during cocaine withdrawal, resulting in both increased LHb neuron excitability and sensitivity to stress-induced reinstatement of cocaine conditioned place preference^50^. This study also reported decreased LHb expression of the vesicular GABA transporter (VGAT) in the LHb following cocaine withdrawal, suggesting that the deficit resulted from reduced GABA concentrations in synaptic vesicles^50^. Although we did not examine VGAT expression following Δ^9^-THC, it is possible that this may represent a common mechanism for altered inhibitory control of LHb excitability as well as the changes in affect that are observed during drug withdrawal.

The impairment of BF-LHb synaptic inhibition was significantly diminished up to 30 d following Δ^9^-THC withdrawal, and this was more apparent during high-frequency activation of BF axons. Thus, although single photostimulation-activated oIPSCs recovered completely 3-7 d after treatment, GABA release by paired-pulse or 10 Hz-stimulation remained significantly dysregulated at 30 d of Δ^9^-THC withdrawal. The 10 Hz-photostimulation frequency activates synapses within the range of alpha/theta band neuronal oscillations that are known to be important in temporally binding activity across brain regions during encoding of fearful and anxiogenic stimuli^51–53^. Moreover, recent studies show that the strength of these oscillations increases in mouse and human LHb in the presence of anxiogenic stimuli^42,54^. Therefore, diminished GABA release probability at these frequencies might be expected to contribute to altered processing of aversive stimuli during Δ^9^-THC withdrawal.

### Time course of LHb CB1R recovery during Δ^9^-THC withdrawal and relationship to impaired GABA release probability

A major consequence of long-term exposure to Δ^9^-THC is the widespread reduction in number and sensitivity of CB1Rs in the brain ^55^. We measured the time course of recovery from reduced CB1R function in LHb and found that the inhibition of BF GABA release by a CB1R agonist was incomplete at 30 d of Δ^9^-THC withdrawal; a time course nearly identical to recovery of GABA release probability at this LHb input. This suggests that the stability of GABA release may depend upon intact CB1R function that is disrupted by repeated exposure to Δ^9^-THC^55^. In support of this idea, tonic CB1R activation by endocannabinoids has been demonstrated at both BF and LPO GABAergic inputs to LHb^25,35^. Additionally, a recent study, using nanoscale localization of CB1Rs in the hippocampus, demonstrated an interaction between CB1R activity and maintenance of GABA release probability, showing that this depends upon CB1R proximity to axon terminal active zones, and that the physical relationship between these sites is disrupted during Δ^9^-THC withdrawal^56^. Thus, ongoing endocannabinoid activation of CB1Rs at axon terminals may promote their interaction with molecular components of neurotransmitter exocytosis to maintain release homeostasis, and extended Δ^9^-THC exposure may disrupt this arrangement.

A recent study demonstrated that tonic firing of LHb neurons was reduced, phasic firing increased, and inhibition of LHb neuron APs by systemic Δ^9^-THC was unchanged during Δ^9^-THC withdrawal in urethane-anesthetized rats^27^. Although the reasons for the disparity between this study and ours are unknown, it is notable that our GCaMP8f measurements were made in awake, freely moving rats during presentation of fearful stimuli. Thus, the changes in spontaneous LHb neuron firing patterns observed in rodents under anesthesia may not reflect their activity in awake subjects where LHb sensory input is intact.

The present data support the idea that the increased excitability of the LHb may contribute to the negative affective states during Δ^9^-THC withdrawal that include dysphoria, enhanced irritability, and increased response to anxiety-provoking stimuli^31,57,58^. As increased excitability of LHb neurons is linked to both depression^26,59–61^, and aversion^10^, it is likely that similar changes in LHb activity during Δ^9^-THC withdrawal will have implications for cannabis withdrawal and CUD in humans. Additionally, since preclinical data indicate that interventional inhibition of LHb neurons can rescue the deleterious effects of LHb neuron hyperexcitability on behavior^26,49^, it is possible that therapeutic interventions designed to limit LHb excitability might be useful to treat several neuropsychiatric conditions in humans, including those associated with drug withdrawal.

## Methods

### Subjects

Male and female Long-Evans rats (postnatal day 42-70), purchased from Charles River Laboratories (Rockville, MD, USA), were used for behavioral experiments. For electrophysiological studies, male Long-Evans rats of the same age were used. Stereotactic injections of viral constructs were performed at postnatal day 52–57, and in vitro electrophysiological experiments were conducted 7–8 weeks later. Animals were housed 2–4 per cage in a temperature- and humidity-controlled facility, had *ad libitum* access to food and water, and were housed under standard lighting conditions (lights on 0600 hr, off 1800 hr). All procedures were designed using the Guide for the Care and Use of Laboratory Animals^62^, and approved by the NIDA-IRP Animal Care and Use Committee. The NIDA-IRP animal facility is accredited by the Association for Assessment and Accreditation of Laboratory Animal Care (AAALAC) International.

### Surgery

Rats were initially anesthetized with isoflurane (4%, in 1 L/min O_2_), then placed in the stereotactic apparatus (Kopf Instruments), and the isoflurane concentration was lowered to 1%-1.5% (0.2 L/min O_2_). Core temperature was maintained at 37 °C using a heating pad. After all surgeries, incisions were closed with absorbable sutures, topical antimicrobial cream was applied to surgical sites, and body temperature was maintained until recovery from anesthesia. Rats were injected with a nonsteroidal anti-inflammatory (meloxicam, 1 mg/kg, s.c.) and then returned to their home cage. Weight and health status were monitored for 3 days after surgery.

### Viral construct injections

For electrophysiological experiments, a 10-µl Hamilton syringe with 35 gauge needle, connected to a UltraMicroPump/SYS-Micro4 controller (WPI) was used to inject 0.7 µl of AAV5-hSynhChR2(H134R)-eYFP (5.2 × 10^12^ virus molecules/ml, University of North Carolina Vector Core), at 100 nl/min, bilaterally into the BF (coordinates from bregma, midline, and level skull, in mm: AP: +1.6; ML: ± 0.8; DV: −6.8), or medial VTA (AP: -5.4; ML: ± 0.2; DV: −8.2, 8° angle). For in vivo fiber photometry, 0.3 µl of pGP-AAV-syn-jGCaMP8f-WPRE (GCaMP8f; Addgene, #162376) was infused at 150 µl/min into the right hemisphere LHb (AP: −3.60, ML: ± 0.60, DV: −5.2). The injection needle was left in place for 5 min and slowly withdrawn to promote diffusion into tissue.

### Optical fiber implants

Immediately after LHb GCaMP8f virus injection, 200 µm core diameter optical fibers (Optogenix, Arnesano, Leece, Italy or Thor Labs, USA) were stereotaxically placed using the same LHb coordinates, with optical fiber tips lowered to 0.1 mm above the virus injection location (i.e., −5.1 mm) and anchored to the skull using 4-stainless steel screws and dental cement.

### Behavioral procedures

Three weeks after surgery, rats were gently handled for 2–3 days, prior to LHb GCaMP8f calcium signal testing. To confirm LHb calcium signal recordings, rats were placed in an operant box (Med-Associates, Fairfax, VT), equipped with two cue lights, a white noise generator, a 2900 Hz tone generator, a house fan, a digital video camera, and a false floor placed over an electrical grid of stainless-steel rods. Two or three brief air puffs were then delivered through a plastic tube positioned with the tip near the rat’s head. Using video monitoring of the rat’s position, air puffs were implemented when the rat neared the plastic tube. Rats showing increased calcium signals in response to air puffs were then randomly assigned to 1 of the 2 treatment groups. Those animals in which acceptable GCaMP signals were not observed were also randomly assigned to treatment groups to be included in the behavioral freezing analysis.

### Fear conditioning, extinction and probe tests

Two days after the final drug or vehicle injections, rats were individually placed in an operant chamber and connected to an optical fiber tether. After 3–5-minutes of acclimation, the first of 10-fear conditioning (FC1) trials was initiated. Each trial consisted of 5 sec of baseline (Pre-CS), followed by 11 sec of CS (tone) presentation, with the last 0.5 sec of tone coinciding with a mild footshock (unconditioned stimulus, US; 0.65 mA) applied through the grid floor. Each trial then continued for an additional 4 sec (20 sec total time), followed by a 60 sec inter-trial interval, prior to beginning the next trial. This procedure was repeated for 10 trials the following day (FC2). One day after FC2, the first of 20 extinction trials consisting of a 5 sec pre-CS period, 11 sec of CS presentation alone, followed by the 4 sec post-CS period, and a 60 sec inter-trial interval was initiated (Ext1). A second extinction session (Ext2) of 20 trials was conducted 1 day following Ext1. Seven days after Ext2, a probe test consisting of 20 trials of CS alone was conducted in an operant chamber in which the metal grid floor was covered by a smooth plexiglass floor that was painted black. Software (Med-PC) was used to control the timing of all behavioral events, and a 28 V/ + 5 V TTL adaptor (Med Associates) was connected to the photometry system to mark event timing. For all behavioral experiments, digital video and GCaMP8f calcium signals were recorded for each trial, and behavior was later scored offline for time spent freezing (the absence of movement) using the ANY-maze software (Stoelting, Wood Dale, IL, USA).

### Fiber photometry procedures

GCaMP8f signals were collected using a commercially available fiber photometry (FP) system (RZ10x, Tucker-Davis Technologies, Alachua, FL, USA). Two light-emitting diodes (LEDs) were used; one at 465 nm to excite GCaMP8f and one at 405 nm (isosbestic control). The timing and intensity of the LED outputs were controlled by the RZ10x system. LED light was filtered and passed through several dichroic mirrors (Fluorescence MiniCube, Doric, Québec, Canada), attached to an optical fiber (200 μm, 0.39 NA, Thorlabs). The MiniCube was also used to direct GCaMP8f emission light from optical fibers to the RZ10x photosensors. The timing and intensity of the light, and recordings of fluorescence signals were set using Synapse software (Tucker-Davis Technologies), with LED intensity set to 30 μW for each channel, measured using an on-board power meter. In order to minimize autofluorescence, cables were photobleached each day prior to initiating recordings. The onset of recording was set to begin 5 sec prior to presentation of a tone channeled through a speaker within the operant chamber.

### Fiber photometry analysis

Fiber photometry data were analyzed off-line using the Photometry Modular Analysis Tool (pMAT version 1.2)^63^. Fluorescence from the signals and isosbestic channels were acquired at 1017 Hz via the TDT RZ10x processor. For analysis, peri-event fluorescence data were collected beginning 5 sec before CS presentation at time = 0 sec and continuing for 4.5 sec after US presentation at time = 10.5 sec (total acquisition time = 20 sec). The *ΔF/F* data were converted to z-scores that were normalized to the signal mean and standard deviation during the first 5 sec of recording, prior to CS presentation. Peri-event plots for the CS-US trials were then constructed around CS presentation and collected for group analyses using z-score peri-event histograms generated by pMAT. All measurements of time are shown relative to CS presentation. Pilot comparisons between *ΔF/F* and z-score data showed no statistical differences in aggregate measurements. Therefore, aggregate z-score data are presented for all comparisons of changes in GCaMP8f signal.

### Brain slice preparation and electrophysiological recording

Brain slices containing the LHb were prepared, and recordings from LHb neurons were conducted using standard procedures^25^. Briefly, rats were deeply anesthetized with isoflurane followed by transcardial perfusion with oxygenated (95% O_2_/ 5% CO_2_), ice-cold (2-4 °C), artificial cerebrospinal fluid (aCSF), (in mM): N-Methyl-D-Glucamine (NMDG) 93, KCl 2.5, NaH_2_PO_4_ 1.2, NaHCO_3_ 30, HEPES 20, glucose 25, sodium ascorbate 5, sodium pyruvate 3, MgCl_2_ 10, CaCl_2_ 0.5 (pH 7.4, 300–310 mOsm). After perfusion, rats were decapitated using a guillotine, and the brain was removed into ice-cold aCSF. Coronal brain slices (250 μm) were then prepared using a vibrating tissue slicer (Leica VT1200S, Heidelberg, Germany). Slices were then incubated in the NMDG-aCSF solution at 34 °C for 10-15 min, then stored in a chamber containing modified aCSF consisting of (in mM): NaCl 109, KCl 4.5, MgCl_2_ 1, CaCl_2_ 2.5, NaH_2_PO_4_ 1.2, NaHCO_3_ 35, glucose 11, HEPES 20, sodium ascorbate 0.4 (pH 7.4, 300-310 mOsm), at room temperature. Unless otherwise indicated, electrophysiological studies were conducted 1 d after withdrawal from long-term Δ^9^-THC or Veh treatment.

### Drug treatment

For behavioral experiments, rats were randomly assigned to 1 of 2 treatment conditions. One group received intraperitoneal (i.p.) injections of Δ^9^-THC (5 mg/kg, emulsified in vehicle consisting of 20% DMSO, 10% Tween80, 70% Saline) once per day for 14 d, whereas the other group received the same number of injections of vehicle (Veh) alone. This Δ^9^-THC treatment regimen in rodents results in plasma levels (50–150 ng/mL)^64^ that are similar to those measured in human marijuana smokers^65–67^. Moreover, numerous studies report both decreased CB1R expression and uncoupling from G-Proteins, as well as behavioral tolerance following similar repeated single-dose treatment paradigms^55^, in both male and female rats^68–70^. For electrophysiological studies, rats were randomly assigned to 1 of 3 treatment groups: 14 d Veh alone (Veh), 14 d Δ^9^-THC alone (THC), or 14 d of Δ^9^-THC, 30 min after i.p. injection of the CB1R antagonist, AM251 (THC + AM).

### Histology

Localization of GCaMP8f-green fluorescent protein expression and optical fiber implants were evaluated in frozen coronal sections of the brain containing the LHb and surrounding regions. Rats were anesthetized with isoflurane and transcardially perfused with phosphate buffer (PB) and 4% paraformaldehyde (PFA). After perfusion, the brain was removed and post-fixed in 4%PFA for 2 hour, then transferred to 30% sucrose until fully submerged. The brain was then frozen in 2-methylbutane at -50 °C and stored at -80 °C until further processing. Coronal sections (50 µm) were cut using a cryostat (Leica CM 3050S, Germany). Sections were then washed 3 ×10 min in PB, followed by incubation in blocking solution containing 0.3 % Triton X-100 (T8787, Sigma, USA), 4 % bovine serum albumin (A9647, Sigma, USA), and Sodium Azide (S2002, Sigma, USA) for 1 hr at room temperature. After blocking, sections were incubated overnight at 4 °C with a primary GFP antibody (mouse anti-GFP-632381, 1:1000, mouse, Takara Bio, USA), diluted in blocking solution. Next, sections were washed with PB (3 × 10 min) and incubated for 2 hour at room temperature with secondary antibody (Alexa Fluor 488, donkey anti-mouse, 715545151, Jackson Laboratories, USA). Sections were washed again with PB (3×10 minutes), mounted on slides, and counterstained with DAPI (DAPI Fluoromount, 1798424, Electron Microscopy Sciences, USA). Images were then acquired and processed using a VS-200 scanner microscope (Olympus, Evident Scientific, USA) and further analyzed by VS-200 desktop software (Version 4.2).

### Data analysis

In electrophysiology experiments, representative evoked traces of synaptic currents are averages of 10 consecutive responses prepared using Clampfit (version 9, Molecular Devices, San Jose, CA, USA), and representative spontaneous synaptic current traces are single 10 sec sweeps collected with WinEDR (v4.1.7, University of Strathclyde, Glasgow, UK). Data spreadsheets were constructed using both GraphPad Prism (v10.4.2, Dotmatics, Boston, MA, USA) and Microsoft Excel.

### Statistics

Statistical analyses were performed using GraphPad Prism, v11.0 (Boston, MA, USA), and data were plotted as mean ± standard error of the mean (SEM) unless otherwise stated. Statistical significance was determined using 1-way or 2-way ANOVAs and paired and unpaired t-tests. Multiple-comparison post hoc tests (Šídák’s) were conducted only after a significant ANOVA interaction or main effect was observed. The Šídák test corrects for multiple comparisons. In all cases, the minimum significance level was set at P < 0.05 using a two-tailed criterion. For statistical analyses in the electrophysiological experiments, n describes the number of neurons from which recordings were made, and the number of rats (n_rats_/n_neurons_) is also reported in the figure captions. A summary of all statistical tests is included in Supplementary Data 1.

### Reagents

DL-AP5, 6,7-Dinitroquinoxaline-2,3-dione (DNQX), DL-2-Amino-5-phosphonopentanoic acid (DL-AP5), (R)-(+)-WIN 55212, and AM251 were purchased from Tocris Bioscience (Bristol, UK). Δ^9^-THC was obtained from the National Institute on Drug Abuse Drug Supply Program (Rockville, MD). Drugs in electrophysiological experiments were dissolved in artificial cerebrospinal fluid (aCSF) and delivered to brain slices via a gravity perfusion system. Drugs were prepared as stock solutions in H_2_O or DMSO and Tween 80 and diluted to the indicated concentrations.

### Reporting summary

Further information on research design is available in the Nature Portfolio Reporting Summary linked to this article.

## Supplementary information

Supplementary Information

Peer Review file

Description of Additional Supplementary Files

Supplementary Data 1

Reporting Summary

## Source data

Source Data

## Data Availability

Source data are provided with this paper.
