# Supplementary material for: Increased encoding of aversive stimuli by lateral habenula neurons during Δ9-tetrahydrocannabinol withdrawal
Source: Nat Commun. 2026 Sep 3;17:10485. doi: 10.1038/s41467-026-77429-9 (PMC13635186; doi:10.1038/s41467-026-77429-9)
Supplement: Supplementary file 1 [file 41467_2026_77429_MOESM1_ESM.pdf]

Data Supplement

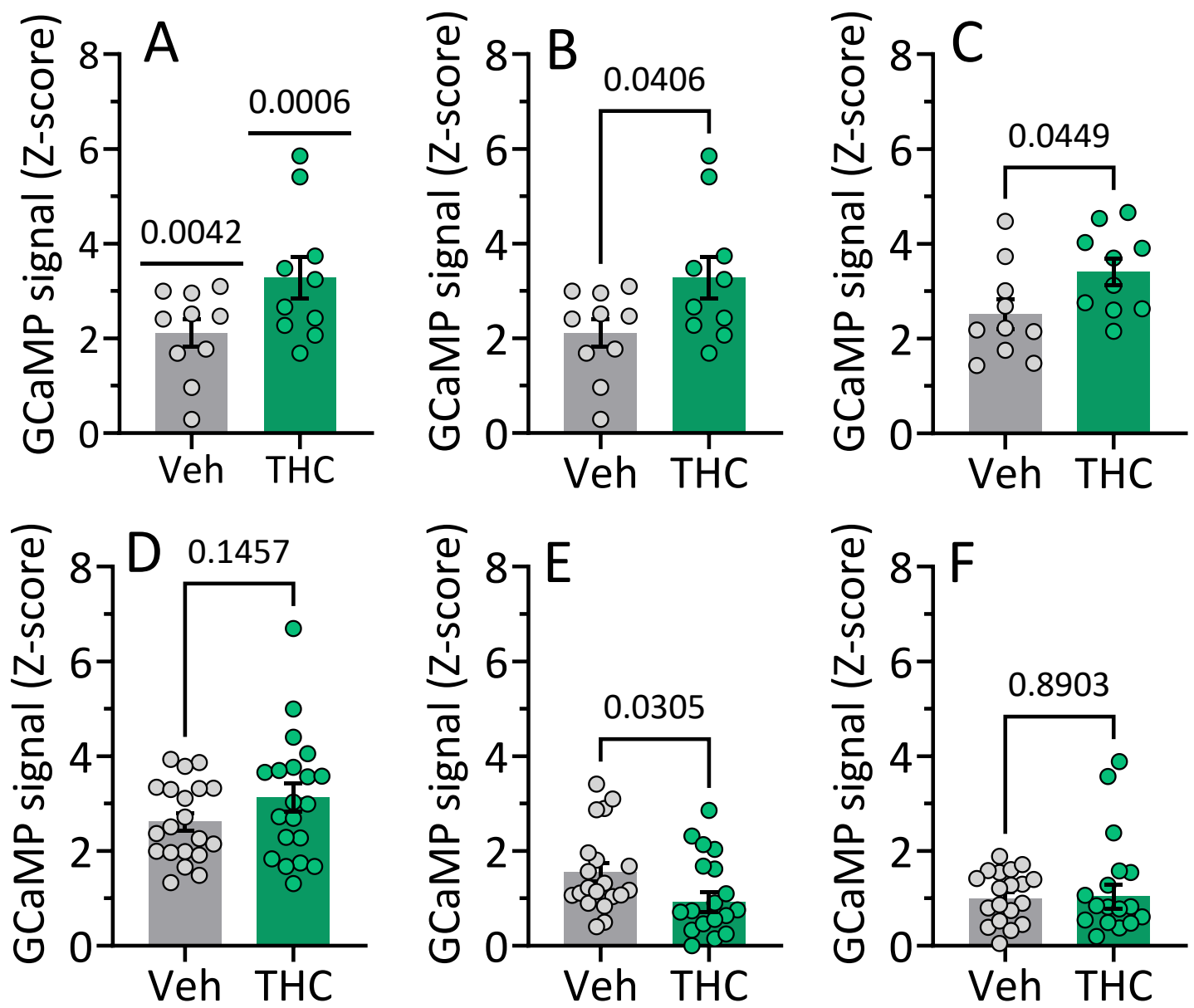

Supplemental Figure 1. Mean LHb GCaMP8f responses (±SEM) to CS pooled across all trials (A-C, 10 trials; D-F, 20 trials) and subjects during Veh and THC withdrawal. **A.** Data from FC1 (Fig. **1D**, bottom) showing result of 1 sample t-tests (vs. Z score =1) demonstrating a significant increase in GCaMP8f signal during CS presentation for both treatment groups (Veh, t=3.801, df=9; THC, t=5.181, df=9). **B.** Same data as panel **A** showing result from an unpaired t-test indicating a significant difference in GCaMP8f signal between groups, across 10 CS-US pairings (n: Veh = 12, Δ^9-THC = 7; t=2.206, df=18). **C.** Data from FC2 (**Fig. 1E**, bottom), pooled across trial and subject, showing result of unpaired t-test indicating a significant difference between withdrawal groups (t=2.156, df=18). **D.** Data from extinction session 1 (Ext1, Fig. **1F**) pooled across trial and subject, showing result of unpaired t-test indicating no significant difference between withdrawal groups (t=1.486, df=38, p=0.1457). **E.** Data from extinction session 2 (Ext2, Fig. **1G**) pooled across trial and subject, showing result of unpaired t-test indicating a significant difference between withdrawal groups (t=2.247, df=38, p = 0.0305). **F.** Data from extinction session 3 probe test (Ext3, Fig. **1H**) pooled across trial and subject, showing result of unpaired t-test indicating no significant difference between withdrawal groups (t=0.1388, df=38, p = 0.8903). All unpaired t-tests 2-tailed.

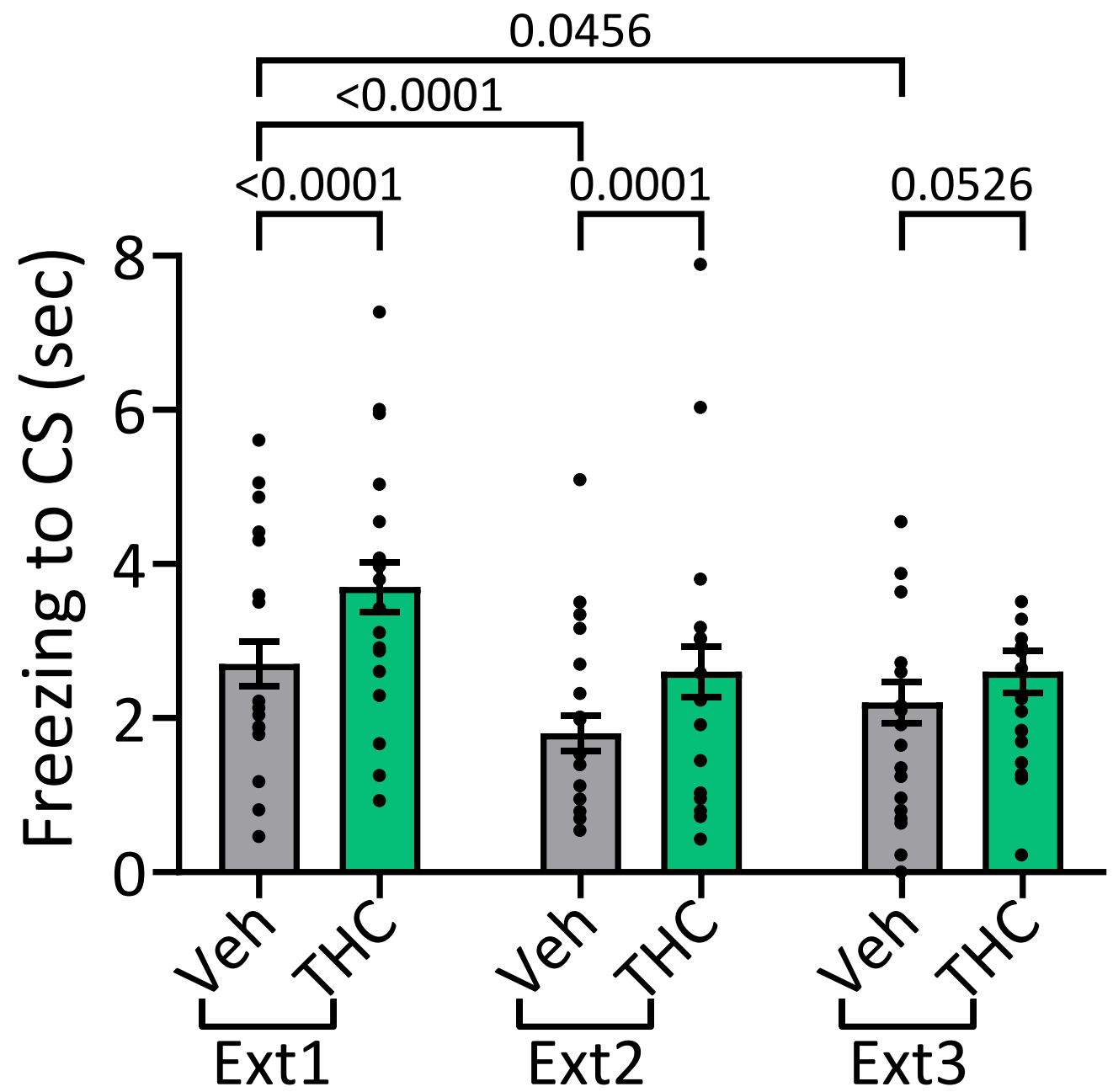

Supplemental Figure 2. Summary of freezing time (mean ± 95% CI) during CS presentation, collapsed across all subjects and trials during extinction sessions 1-3, after withdrawal from 14-day vehicle or Δ^9-THC treatment (Veh, n = 19; THC, n = 18). Two-way ANOVA, interaction = $F_{2, 2154} = 2.194$, p=0.1118; treatment main effect, $F_{1, 2154} = 37.92$, $p < 0.0001$; extinction session main effect = $F_{2, 2154} = 26.32$, $p < 0.0001$. The data show that rats withdrawn from Δ^9-THC showed increased freezing during Ext1 and Ext2, compared to Veh withdrawn rats, and that extinction progressed across sessions in all groups (p values, Šídák's post hoc test).

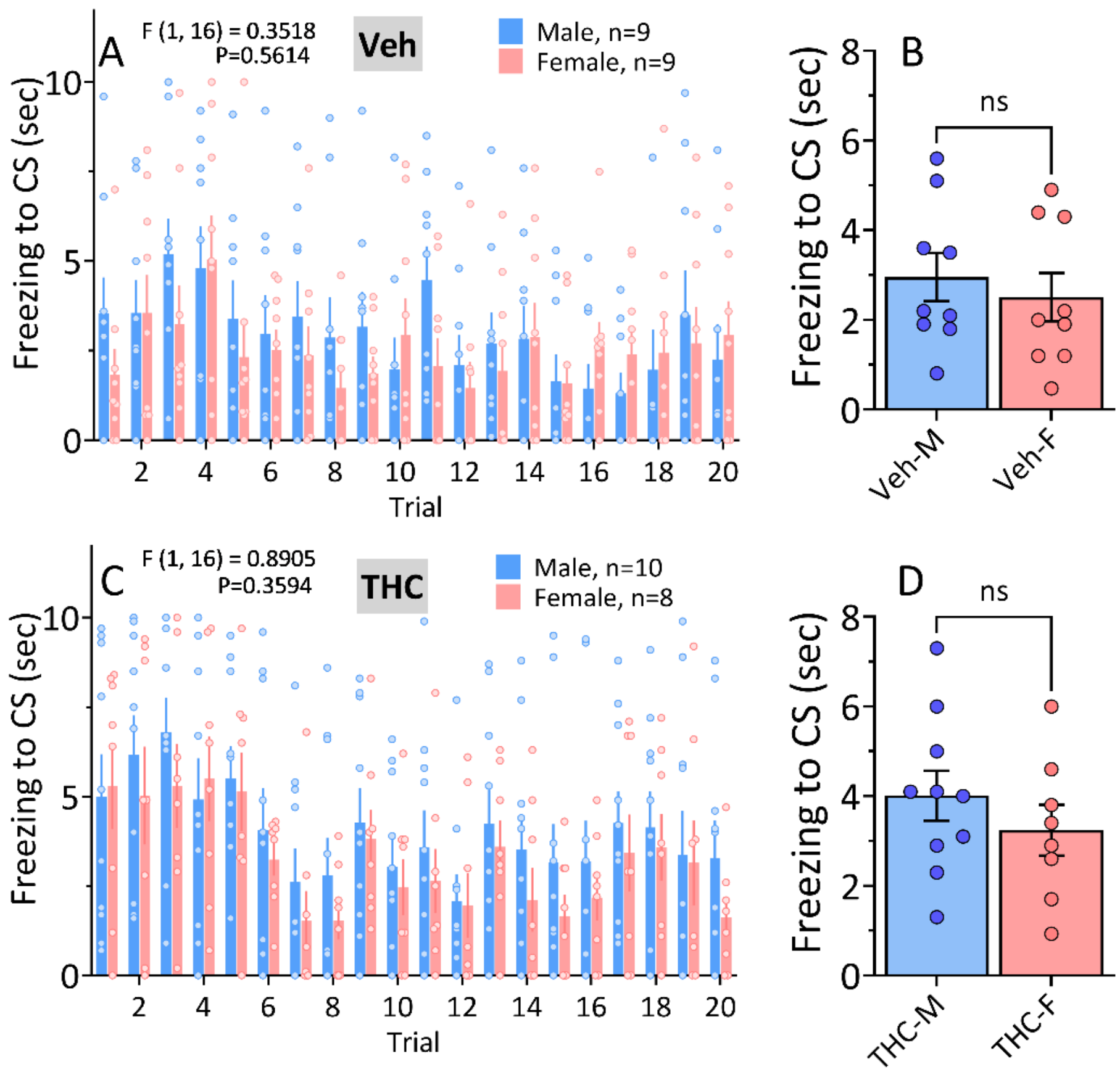

Supplemental Figure 3. Absence of sex differences in freezing behavior to CS presentation in fear conditioned rats during Ext1. **A.** Comparison of male and female rat freezing times across 20 trials of CS only presentation during Veh withdrawal. The number of subjects in each group and result of the statistical comparison using a 2-way RM-ANOVA (main effect of sex) are shown. **B.** Data from **A** collapsed across all trials and subjects and analyzed by unpaired t-test (t_{16}=0.5915, p = 0.5625). **C.** Comparison of male and female rat freezing times across 20 trials during THC withdrawal. The number of subjects in each group and result of the statistical comparison using a two-way RM-ANOVA (main effect of sex) are shown. **D.** Data from **C** collapsed across all trials and subjects and analyzed by unpaired t-test (t_{16}=0.9543, p = 0.3541). All bars, (mean ± SEM); ns = not significant, unpaired t-test.

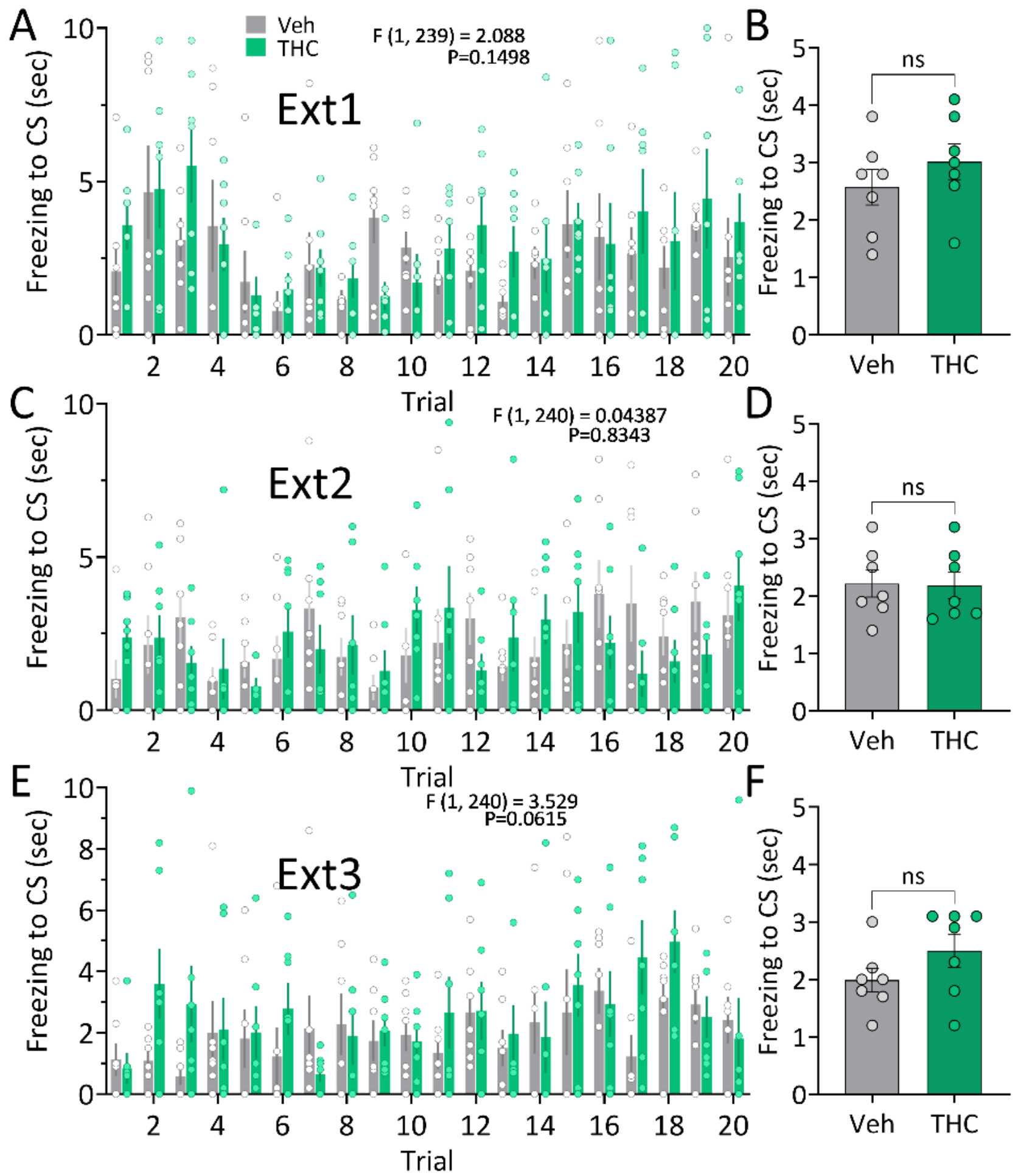

Supplemental Figure 4. Freezing behavior to CS presentation does not differ between THC and Veh withdrawn rats, following unpaired US-CS training. During conditioning footshocks were delivered at random unpredictable intervals (20-90 sec) after presentation of the CS (10 sec tone) ended, for 2 daily, 10-trial conditioning sessions. The extent of conditioning was assessed by measuring freezing during CS presentations across extinction sessions 1-3. **A, C, E.** Trial-by trial mean (±SEM) time spent freezing in Veh or THC withdrawal groups. Two-way ANOVA of Ext1-3 sessions revealed no significant interaction or main effects of treatment. ANOVA treatment main effect result is shown. **B, D, F.** Bar graphs showing data in **A, C,** and **E** collapsed across trials for each extinction session (mean ±SEM). All comparisons not significant (ns) with 2-tail unpaired t-test. The data indicate that the significantly slower extinction of the freezing response observed in THC withdrawn rats (**Figs. 1I and 1J**) in paired CS-US experiments did not result from a generalized heightened response to the conditioning stimuli (n = 7 male rats per group).

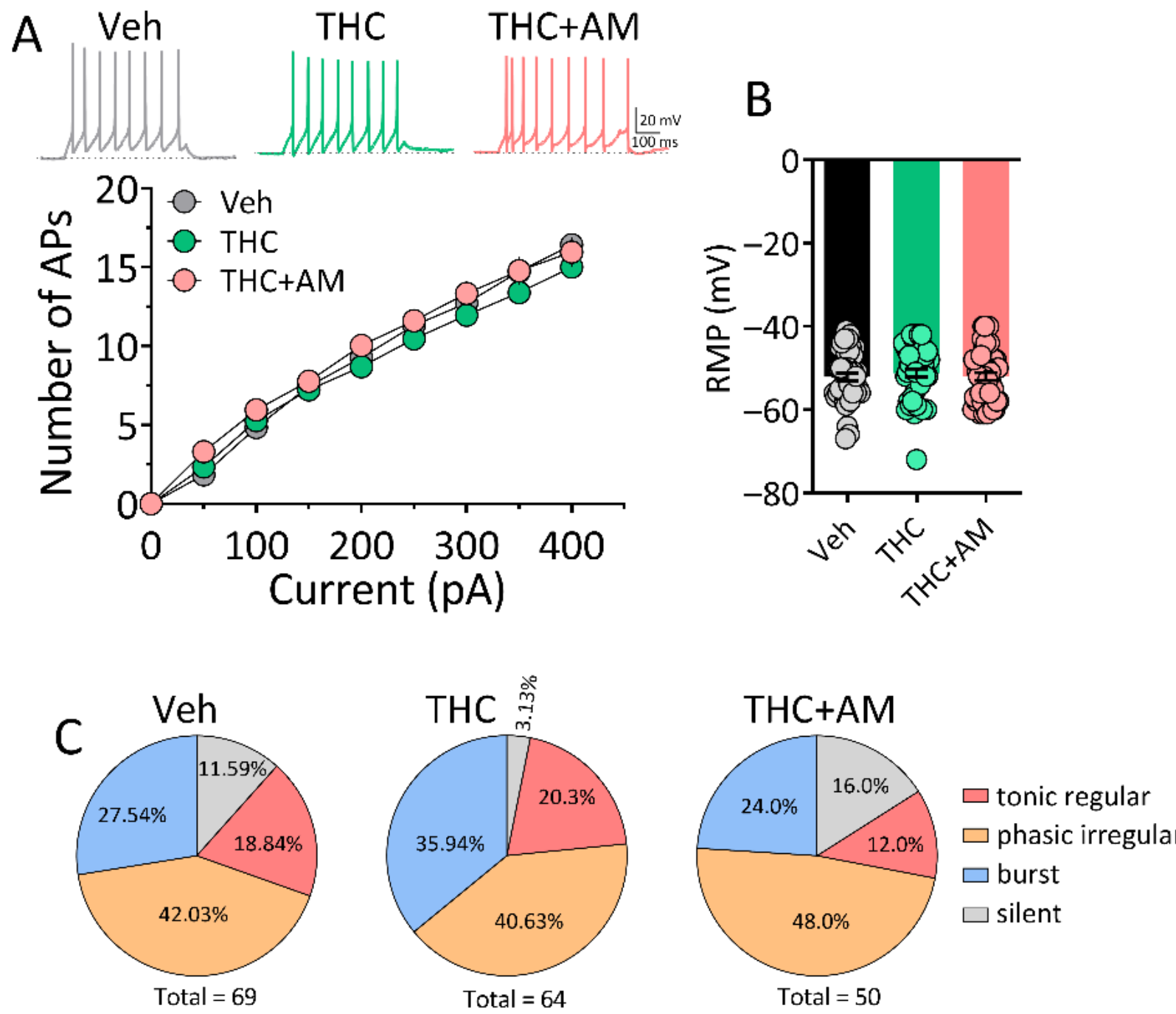

Supplemental Figure 5. No change in active or passive membrane properties of LHb neurons at 1d Δ^9-THC withdrawal. **A**. Unaltered relationship between injected current amplitude and AP number in all 1-d withdrawal treatment groups. **B**. No change in mean (±SEM) resting membrane potential across all withdrawal treatment groups. **C**. No change in spontaneous firing patterns of LHb neurons during withdrawal from all treatment conditions *in vitro* (X^2_6 = 8.193, p = 0.2243. n-rats/n-neurons: **A-B**, Veh = 10/26, THC = 11/26, THC+AM = 10/23).

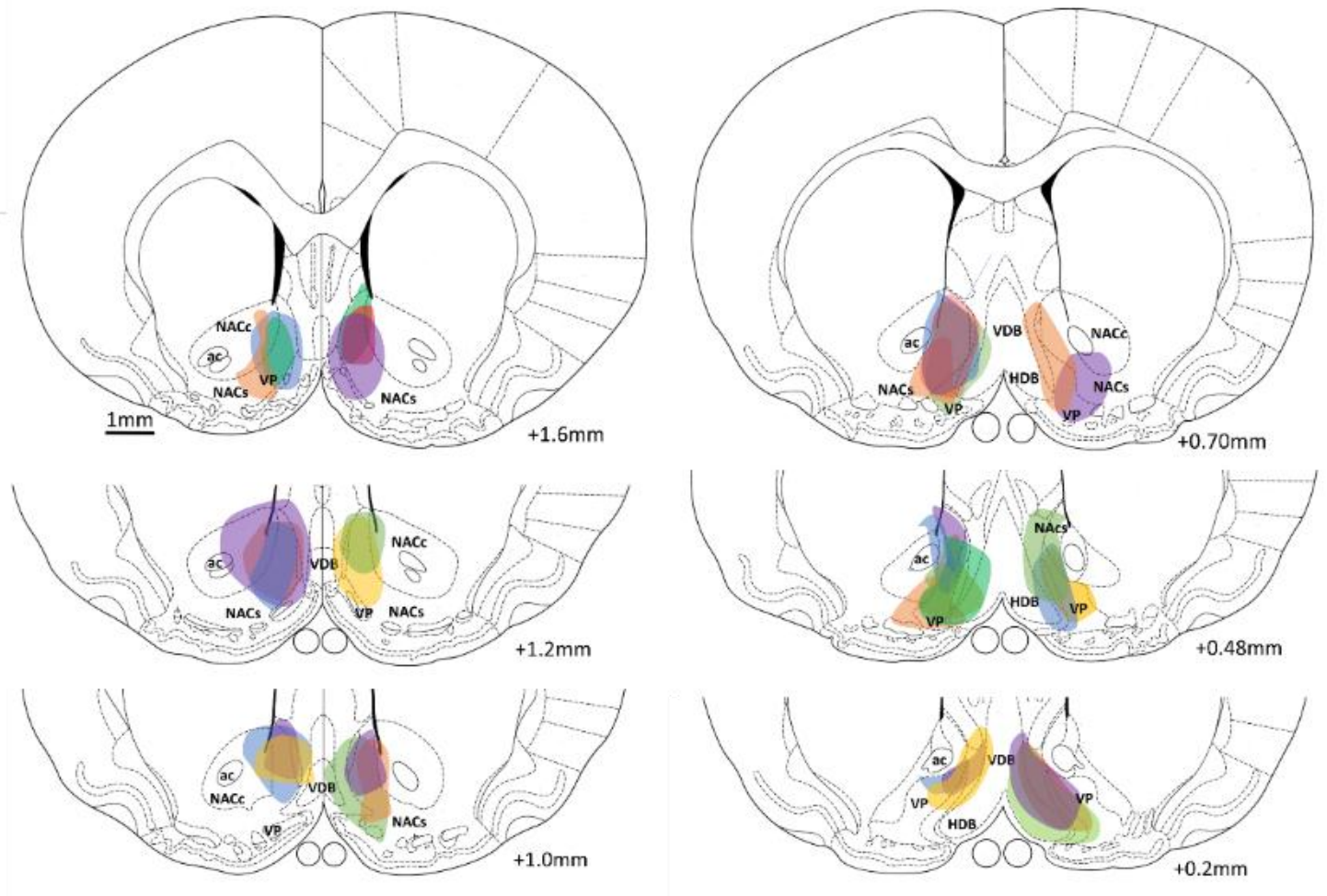

Supplemental Figure 6. Histological reconstructions of channelrhodopsin-2 virus (AAV5-hSyn-ChR2-eYFP) eYFP fluorescence near injection sites used to verify bilateral coordinates for *in vitro* optogenetic experiments (n= 16 rats). Injection coordinates targeted an area comprised of the rostral VP, caudal NAcs, as well as portions of the diagonal band, as previously described [1]. eYFP expression extend from +0.2mm to +1.6mm anterior to bregma [2]. The extent of virus expression is depicted as shades of color across several subjects and localizations in each section. Specific colors do not represent data from individual animals across sections. Abbreviation: ac, anterior commissure; HDB, horizontal diagonal band; NACc, nucleus accumbens core; NAcs, nucleus accumbens shells; VDB, vertical diagonal band; VP, ventral pallidum. Atlas plates reproduced with permission, from The rat brain in stereotaxic coordinates. Paxinos and Watson, 4th Ed., Elsevier, 1998, license ID: 1761405-1.

References

1 Hwang EK, Zapata A, Hu V, Hoffman AF, Wang HL, Liu B, et al. Basal forebrain-lateral habenula inputs and control of impulsive behavior. Neuropsychopharmacology. 2024;49(13):2060–68.

2 Paxinos G and C. Watson The Rat Brain in Stereotaxic Coordinates, 4th Edition. Academic Press: San Diego, USA; 1998.
