# Supplementary material for: Increased encoding of aversive stimuli by lateral habenula neurons during Δ9-tetrahydrocannabinol withdrawal
Source: Nat Commun. 2026 Sep 3;17:10485. doi: 10.1038/s41467-026-77429-9 (PMC13635186; doi:10.1038/s41467-026-77429-9)
Supplement: Supplementary file 2 [file 41467_2026_77429_MOESM2_ESM.pdf]

nature portfolio

Peer Review File

Increased encoding of aversive stimuli by lateral habenula neurons during Δ9-tetrahydrocannabinol withdrawal

Corresponding Author: Dr Carl Lupica

This file contains all reviewer reports in order by version, followed by all author rebuttals in order by version.

Version 0:

Reviewer comments:

Reviewer #1

(Remarks to the Author)
The study explores the involvement of the lateral habenula (LHb) in cannabis withdrawal, particularly how it impacts fear responses and neural activity. Researchers examined withdrawal from Δ9-tetrahydrocannabinol (Δ9-THC) by measuring LHb calcium activity with fiberphotometry during fear conditioning and conducting in vitro electrophysiology. They found that LHb neurons exhibited heightened responses to footshock and tone presentation, with withdrawal-associated rats showing increased fear-induced freezing behavior. Electrophysiological analysis revealed an elevated excitatory-inhibitory (E-I) ratio due to impaired synaptic GABA release. Specifically, GABA release from basal forebrain (BF) inputs to LHb was disrupted, while input from the ventral tegmental area remained unaffected. Notably, recovery of BF-LHb GABA transmission and CB1R desensitization remained incomplete after 30 days of withdrawal. The authors conclude that cannabis withdrawal-induced negative affect is linked to LHb hypersensitivity to aversive stimuli, reinforcing the encoding of environmental stress cues.
Several critical points must be addressed before further evaluation can take place. Additional experiments and analyses are required to ensure the robustness and consistency of the findings.
A general concern is the use of both male and female between 42 and 70 days old across the study but only male rats of the same age for electrophysiological recordings. Thus, detailed statistical tables must be provided with separate reports for male and female. If male and female are proven statistically identical then and only then can they be mixed in the results. An explanation as to why mixed sexes were used in all experiments and only male of the same age for electrophysiological experiments must still be provided.

FIGURE 1:

-Why was a constant THC dosage used? THC quickly desensitizes CB1R and downstream signaling. Could the authors clarify why an escalating schedule was not preferred?
-The experimental timeline raises significant concerns and must be clarified. Specifically, why were fear conditioning sessions (FC1–2) conducted 48- and 72-hours post-withdrawal, while extinction trials were carried out between the third and fourth day of withdrawal? This discrepancy demands an explanation—are the animals still experiencing withdrawal symptoms during this period?
-The authors present freezing behavior exclusively during extinction trials. Strikingly, WD (4D) rats exhibit significantly higher freezing at the onset of extinction, gradually aligning with control levels by trial 10. This pronounced initial freezing suggests a stronger acquisition of fear responses within the withdrawal group, raising critical questions about how this aligns with the authors' overarching hypothesis.
-Given that freezing behavior is initially higher in WD rats, it seems that extinction may be more pronounced in this group compared to the sham group. To validate this observation, the authors should plot freezing data from EXT1 and EXT2 on a single graph and apply appropriate statistical analyses to determine whether the sham group also demonstrates extinction. Normalizing freezing levels during the initial trials (e.g., trials 1–3) could provide additional clarity. Moreover, a key concern arises in (1F), where sham mice do not exhibit freezing extinction in response to the CS.
-Freezing behavior should be shown longitudinally, from FC1 and FC2 through EXT1, EXT2, and the probe session.
-Although the LHb reliably responds to shocks, the z-score data for the CS (Supplementary Figure 1) are unclear as some mice in the sham group show no CS response. The authors should 1/ include picture(s) of GCaMP expression in the LHb to confirm consistent GCaMP expression and LHb targeting, and 2/ plot and test for the correlation between CS and US responses.

-In many instances, the largest differences appear during the initial trials. This may indicate a "startle response" rather than a genuine CS-US association. Additional experiments with unpredictable shocks and CS tones will clarify this.
-Additionally, since LHb responses to shocks are stable over time in physiological condition but vary across trials, the authors must represent average responses across five trials (Figure 1D–E), rather than plotting trial-by-trial data.

FIGURE 2:

The data presented are compelling and well-executed, but a comprehensive understanding of withdrawal-induced plasticity requires chronic IPSC and EPSC recordings. To achieve more robust statistical analysis, it is advisable to plot sIPSC and sEPSC frequency and amplitude as cumulative probability plots, employing methods such as the Kolmogorov-Smirnov (KS) test.
There is concern regarding the withdrawal period selected for these experiments. Specifically, recordings were conducted one day after the cessation of THC administration, while in a previous figure, data were obtained two days post-withdrawal. This inconsistency raises questions about the reliability of the findings. How can one be confident that the excitatory-inhibitory imbalance remains as pronounced after 48 hours of withdrawal?
Additionally, there is concern about how individual variability has been accounted for. The individual unit of analysis should be the rat rather than the recorded neuron. Therefore, all statistical analyses must be reevaluated to ensure methodological rigor and accurate interpretation of the data.

FIGURE 3:

To fully assess the excitatory-inhibitory balance within LHb circuits, it is essential to conduct experiments measuring excitatory-inhibitory (E/I) ratios and paired-pulse ratios specifically for excitatory inputs.
One major challenge in studying the BF is its anatomical complexity, as it comprises multiple regions, including the ventral pallidum and nucleus accumbens. Research shows that many inputs to the LHb can release glutamate and GABA, either as corelease or from parallel inputs. Thus, targeting the BF is not precise enough. To determine whether the observed effects are consistent across BF's subregions, additional targeted experiments must be performed. Specifically, to identify the key input region responsible for LHb modulation, channelrhodopsins should be expressed selectively in either the VP or the nucleus accumbens.
Furthermore, employing two distinct opsins that respond to different wavelengths would allow for selective and simultaneous stimulation of both input pathways. This approach would enable researchers to test the hypothesis that effective LHb modulation requires cooperative input from both regions rather than isolated activity from a single source. These experiments are crucial to refining our understanding of the network dynamics underlying LHb function during withdrawal states.

Reviewer #2

(Remarks to the Author)

This is a highly compelling and meticulously executed study that significantly advances our understanding of the neurobiological underpinnings of cannabis withdrawal. The authors employ a sophisticated and complementary array of techniques—fiber photometry, in vitro electrophysiology, and ex vivo optogenetic experiments—to dissect the circuit-specific alterations in the lateral habenula (LHb) during Δ9-THC withdrawal in rats. Their findings provide robust and novel insights into how a dysregulation of endocannabinoid (eCB) signaling within specific GABAergic inputs to the LHb contributes to the negative affective states associated with cannabis withdrawal. The rigor of the experimental design and the clarity of the results make this a truly outstanding contribution to the field.

The central aim of this work was to unravel how cannabis withdrawal alters LHb function and its interactions with key GABAergic afferents, ultimately impacting fear-related behavior. Addressing a critical gap in the literature regarding the neurobiological consequences of cannabis withdrawal on brain function at the circuit level, the study provides strong evidence that Δ9-THC withdrawal leads to heightened LHb neuronal and population activity, which can directly contribute to increased behavioral sensitivity to aversive stimuli.
Specifically, the authors demonstrated using fiber photometry that withdrawn rats exhibited enhanced GCaMP calcium responses in the LHb during Pavlovian fear conditioning. This indicates a stronger encoding of aversive associations, consistent with the heightened negative affect observed during withdrawal from chronic cannabis use. This exaggerated activity was also observed during extinction sessions, concomitant with an increased freezing response to the conditioned stimulus, suggesting a persistent alteration in fear processing.

Mechanistically, the study precisely identified a selective impairment of inhibitory synaptic transmission within the LHb during withdrawal. Through in vitro electrophysiology, the authors demonstrated a significant reduction in the frequency of spontaneous inhibitory postsynaptic currents (sIPSCs) in LHb neurons from withdrawn rats. Crucially, this reduction was prevented by co-administration of the CB1 receptor (CB1R) antagonist AM251, strongly implicating the involvement of CB1Rs in this impairment of inhibitory control of LHb.
The ex vivo optogenetic experiments further refined this understanding, revealing that this GABAergic deficit was specific to inputs originating from the basal forebrain (BF), encompassing projections from the nucleus accumbens shell and ventral pallidum. In stark contrast, inhibitory inputs derived from the ventral tegmental area (VTA) to the LHb remained unaffected by

Δ9-THC withdrawal. This input-specific dysregulation is a particularly significant finding, as it highlights a discrete circuit mechanism underlying the observed behavioral changes. The authors conclusively showed that the ability of presynaptic CB1Rs to inhibit GABA release is functionally compromised at BF inputs to the LHb for at least 30 days post-withdrawal, leading to a functional shift towards excitation and increased LHb excitability.
In sum, this study provides robust evidence that the negative affective state observed during cannabis withdrawal is underpinned by a specific, long-lasting dysregulation of eCB modulation of GABAergic transmission from the basal forebrain to the LHb, resulting in an excitatory/inhibitory (E/I) imbalance within the LHb that can contribute to altered emotional processing and enhanced negative affect.

The study is exceptionally well-done and highly rigorous, providing compelling evidence for a novel mechanism in cannabis withdrawal. I have only three minor comments for the authors to consider:
1. Methods Section Clarity (Electrophysiology & Optogenetics): I noted that the "Methods" section appears to lack specific subsections detailing the procedures for electrophysiological and optogenetic recordings. For the sake of clarity and reproducibility, it is crucial to include dedicated subsections outlining the precise protocols, equipment, software, and analysis methods used for both the in vitro electrophysiology and the ex vivo optogenetic experiments. This would enhance the manuscript's completeness and allow other researchers to replicate these technically challenging experiments.

2. Spontaneous Cell Firing Pattern Analysis: While the authors state that Δ9-THC withdrawal did not significantly alter the overall pattern of spontaneous cell firing (Fig. S1C, Chi-square value = 5.670, $p = 0.1272$), I am curious about the specifics of this analysis.

Could the authors clarify their criteria for classifying patterns of irregular versus regular tonic activity? A precise definition would be beneficial.
Furthermore, I am curious if combining the categories of "tonic" (both regular and irregular) and "bursting" cells and comparing this proportion to "silent" cells would reveal a significant shift in the proportion of spontaneously active versus silent neurons. Given the profound changes in E/I balance and excitability observed elsewhere, a subtle shift in the overall proportion of active vs. silent cells might be masked by the current categorization, and exploring this alternative grouping could provide additional insight into the general excitability state of LHb neurons post-withdrawal.

3. Role of Astrocytes in LHb Hyperexcitability: The study provides compelling evidence for neuronal mechanisms underlying LHb hyperexcitability. However, the rich literature on eCB-regulation of astrocytes, particularly within the LHb, suggests that these glial cells could also contribute to the heightened LHb activity observed during cannabis withdrawal. Given that CB1Rs are expressed in LHb astrocytes and their activation can modulate synaptic transmission and calcium signaling as well as negative affective states, a discussion of this possibility would be a valuable addition. Considering how astrocytic eCB signaling might be altered during withdrawal and subsequently influence neuronal function could provide a more comprehensive picture of the circuit-level changes.

Reviewer #3

(Remarks to the Author)
In this work, the authors put forward the hypothesis that the lateral habenula contributes to aspects of cannabis withdrawal. Experimentally, the authors evaluated this by mean of photometric measurement of calcium responses during fear conditions in rats that experienced withdrawal from Δ9-THC. The main finding is that aversive footshock and predictive CS lead to GCaMP8f signal increases in withdrawal compared to vehicle animals. This higher calcium dynamics was linked to increased freezing during CS presentation after cannabis vapor exposure. Using slice physiology, the authors reported changes in E/I balance stemming from reduced synaptic inhibition of LHb neurons – these effects were CB1R dependent using specific pharmacology.

This study expands on the notion that plastic adaptations in the lateral habenula underscore aspects of drug withdrawal and bring new insights on cannabis withdrawal which at the moment was lacking. The combination of different approaches is elegant and the question is timely and certainly of interest for the field.

Major concerns.
1. The calcium dynamics elicited after shock and CS raise some question. The representative traces indicate a rundown across trials. This is visible in the Ext sessions and partly also in the FC sessions. Furthermore, it is unclear why only CS trial 4 and 6 are larger. How is the distribution of responses across mice? Do the authors see systematically such CS driven larger transients only on these trials? This should be further quantified and clarified in the text.

When the authors assess freezing behaviors in panel K, freezing seems augmented only on certain trials, but during those trials CS dynamics in LHb seems not really altered. The authors should provide some discussion around this point and if possible a manipulation of the circuit to understand the link between function and behavior.

2. The question on whether such activity dynamics plasticity in fear conditioning is a feature of cannabis withdrawal or encompasses other drugs should be tested.

3. The authors discuss the importance of basal forebrain inputs onto LHb mentioning regions of the pallidum and accumbens. However it remains unclear where these axonal innervation comes from. In addition the lack of injections site visualisation and innervation in the habenula is lacking. This portion of the study deserves a much thorough dissection to define the inputs organisation as BF remains a vague term encompassing large territories with different behavioral relevance.

4. The comparison between BF and VTA is interesting. However, a rationale for choosing the VTA remains elusive. GABA inputs onto the habenula emerge from the GPi in the basal ganglia, as well as the septum. Is a comparison limited to the VTA appropriate? The authors should experimentally test this concern.

5. Presynaptic GABA release is controlled by different machineries. One is the endocannabinoid system, but others relie on GABAb receptors or opioid receptors. The authors could test some controls to understand if other machineries adapt as well during cannabis withdrawal.

Version 1:

Reviewer comments:

Reviewer #1

(Remarks to the Author)
The authors have adequately and thoroughly addressed my concerns. The additional experiments and analyses are greatly appreciated. Congratulations on a job well done.

Reviewer #2

(Remarks to the Author)
The authors have comprehensively addressed all of the issues raised in my previous assessment, and I have no further comments. The revised manuscript is excellent. This work is highly significant, and I congratulate the authors for accomplishing such an outstanding study, particularly given the substantial difficulties they endured during the government shutdown.

Reviewer #3

(Remarks to the Author)
The authors have addressed my concerns. The outcome of this work is of excellent quality further supporting evidence for the role of habenular adaptations in drug withdrawal processes. I support publication in its current form.

Open Access This Peer Review File is licensed under a Creative Commons Attribution 4.0 International License, which permits use, sharing, adaptation, distribution and reproduction in any medium or format, as long as you give appropriate credit to the original author(s) and the source, provide a link to the Creative Commons license, and indicate if changes were made.
In cases where reviewers are anonymous, credit should be given to 'Anonymous Referee' and the source.
The images or other third party material in this Peer Review File are included in the article's Creative Commons license, unless indicated otherwise in a credit line to the material. If material is not included in the article's Creative Commons license and your intended use is not permitted by statutory regulation or exceeds the permitted use, you will need to obtain permission directly from the copyright holder.
To view a copy of this license, visit https://creativecommons.org/licenses/by/4.0/

Response to Reviewer Comments

All responses to reviewer comments are presented in bold italics and all major revisions within the manuscript are indicated by gray highlights.

The authors would like to thank the reviewers for their helpful and constructive comments on our manuscript. We have made several changes and have added experiments in-line with these comments.

Reviewer #1 (Remarks to the Author):

The study explores the involvement of the lateral habenula (LHb) in cannabis withdrawal, particularly how it impacts fear responses and neural activity. Researchers examined withdrawal from Δ9-tetrahydrocannabinol (Δ9-THC) by measuring LHb calcium activity with fiberphotometry during fear conditioning and conducting in vitro electrophysiology. They found that LHb neurons exhibited heightened responses to footshock and tone presentation, with withdrawal-associated rats showing increased fear-induced freezing behavior. Electrophysiological analysis revealed an elevated excitatory-inhibitory (E-I) ratio due to impaired synaptic GABA release. Specifically, GABA release from basal forebrain (BF) inputs to LHb was disrupted, while input from the ventral tegmental area remained unaffected. Notably, recovery of BF-LHb GABA transmission and CB1R desensitization remained incomplete after 30 days of withdrawal. The authors conclude that cannabis withdrawal-induced negative affect is linked to LHb hypersensitivity to aversive stimuli, reinforcing the encoding of environmental stress cues.

Several critical points must be addressed before further evaluation can take place.

Additional experiments and analyses are required to ensure the robustness and consistency of the findings.

A general concern is the use of both male and female between 42 and 70 days old across the study but only male rats of the same age for electrophysiological recordings. Thus, detailed statistical tables must be provided with separate reports for male and female. If male and female are proven statistically identical then and only then can they be mixed in the results.

Analysis of the fear conditioning data showed no significant differences between male and female rats in Veh or THC withdrawal conditions. An analysis of sex differences was not performed the GCaMP data because this portion of the study was not powered for such a comparison.

The lack of sex differences in fear conditioning extinction are now reported in Results, and a figure added to the data supplement (Fig. S3). Additionally, we have included a detailed statistical summary for all data as a supplemental spreadsheet.

An explanation as to why mixed sexes were used in all experiments and only male of the same age for electrophysiological experiments must still be provided.

This work originates from a NIH USA federal government laboratory. Throughout the course of these experiments the laboratory has had to deal with prolonged US government shutdowns, severe budget restrictions, firings of support personnel, and a general inability to expend funds and place orders as needed. For example, additional rats of appropriate age and sex were ordered to address reviewer comments and arrived just prior to the September 2025 US government wide 6-week closure. However, after the shutdown, when our work could begin again, we found that these animals had become too old and large for inclusion in our experimental groups. This required placement of new requests for subjects and additional delays because of the limited numbers of procurement personnel available to process orders. Because of these issues we have had to largely rely upon wildtype LE rats produced within our breeding facilities, with limits on the numbers of animals of the appropriate age and sex for our experiments. Thus, mixed sexes were used in fear conditioning experiments because these animals were available from our animal facility in sufficient numbers at the appropriate age. However, for the electrophysiological experiments few females were available and the decision to use only males was made. For these studies we made note of the observations by several studies reporting profound desensitization of CB1R function and downregulation of CB1R number throughout the brain following long-term THC exposure in adult male and female rats (Burston et al., 2010; Farquhar et al., 2019; Rubino et al., 2008). Thus, we are confident that these changes occur in both sexes. We have now included this information in the "Drug Treatment" section of Methods.

FIGURE 1:

-Why was a constant THC dosage used? THC quickly desensitizes CB1R and downstream signaling. Could the authors clarify why an escalating schedule was not preferred?

The dose of Δ^9 -THC used in our experiments (5 mg/kg) was chosen because it results in blood concentrations in rodents (50–150 ng/mL) (Nguyen et al., 2016) that are similar to those measured in human marijuana smokers (Huestis et al., 1992). However, it is important to also note that the blood plasma concentration of Δ^9-THC reported in this latter study likely underestimates human plasma levels that are relevant to contemporary widely available strains of cannabis that contain up to 4-fold higher levels of Δ^9-THC than that used in this study (McLaren et al., 2008).

With further relevance to tissue levels of Δ^9-THC, we reported previously that the phytocannabinoid is not detectable in rodent brain tissue with liquid chromatography–mass spectrometry (LCMS), 24 hours after 7, once per day, i.p. injections of a higher dose (10 mg/kg) (Hoffman et al., 2007). Therefore, the dose chosen in our study roughly approximates that which produces similar plasma levels in human chronic cannabis users, and at 24 hours of withdrawal brain levels of the phytocannabinoid are negligible.

With regard to our use of a non-escalating dosing paradigm, the utility of using an escalating paradigm has, to our knowledge, not been empirically demonstrated with comparison to non-escalating paradigms. Also, the majority of behavioral and cellular studies reporting Δ^9-THC tolerance and dependence have used single doses across time (Sim-Selley, 2003), and numerous studies report decreased CB1R expression and coupling to G-Proteins, as well as behavioral tolerance following similar repeated single-dose treatment paradigms (Sim-Selley, 2003). In general, these results have been remarkably similar with regard to behavioral, cellular, and receptor changes across laboratories using these constant Δ^9 -THC dosing paradigms. Finally, the data that we present in the current manuscript shows that the non-escalating treatment paradigm produced marked CB1R desensitization

and impaired synaptic inhibition in the LHb, suggesting that long-term treatment with the single daily-dose paradigm was effective.

To address the questions raised by the reviewer, we have added a brief rationale for the dosing paradigm used in our study in the Methods section.

-The experimental timeline raises significant concerns and must be clarified. Specifically, why were fear conditioning sessions (FC1–2) conducted 48- and 72-hours post-withdrawal, while extinction trials were carried out between the third and fourth day of withdrawal? This discrepancy demands an explanation—are the animals still experiencing withdrawal symptoms during this period?

This timeline was adopted to permit a longitudinal within subjects model. Additionally, FC1 began 48 hr after the last Veh/THC injection to ensure no residual brain levels of THC that might confound interpretation of withdrawal-related effects. For obvious reasons, extinction trials could only be conducted after FC training. Based upon the lack of reversal of the synaptic changes observed during withdrawal (decreased CB1R sensitivity and synaptic impairment) 3 days after the last Veh/THC injection, we reason that withdrawal is ongoing during the extinction sessions. However, as the probe test occurred 12 days after the final injection, when CB1R sensitivity and the synaptic impairment showed some reversal, the magnitude of withdrawal had likely diminished, suggesting that the strength of the memory trace was assessed independently of withdrawal.

-The authors present freezing behavior exclusively during extinction trials. Strikingly, WD (4D) rats exhibit significantly higher freezing at the onset of extinction, gradually aligning with control levels by trial 10. This pronounced initial freezing suggests a stronger acquisition of fear responses within the withdrawal group, raising critical questions about how this aligns with the authors' overarching hypothesis.

As the reviewer indicates, higher levels of freezing to the CS during the initial extinction trials may indicate a stronger conditioned fear response in the THC withdrawn subjects, compared to vehicle controls, and this diminishes as extinction progresses. Thus, the initial fear response (freezing) during extinction is stronger in the THC treatment group, but extinction itself progressed with additional trials. The increased freezing time in the THC withdrawal group can be interpreted as an increased resistance to extinction, the development of a stronger fear memory, or perhaps an increased general reactivity to stimuli. Our central hypothesis was that an elevated negative emotional state during THC withdrawal would facilitate fear learning (i.e. a larger fear response to the CS). Therefore, the data support this overarching hypothesis.

However, to further address the possibility of a generalized increased sensitivity to novel stimuli or increased resistance to extinction in the THC withdrawal group, we have added an additional experiment in which the CS was not explicitly paired with footshock and find that the THC withdrawn animals do not show increased freezing to the CS alone during extinction (described below). Therefore, in light of this, and since the withdrawn rats in our original experiment showed increased LHb responses to both the CS and US, and increase increased freezing responses during extinction, we conclude that these rats developed a stronger fear memory. The results from the additional "unpaired" experiment is now described in Results and presented in the Supplemental data section (Fig. S4). Additionally, we address these issues in the revised discussion section of the manuscript.

-Given that freezing behavior is initially higher in WD rats, it seems that extinction may be more pronounced in this group compared to the sham group. To validate this observation, the authors should

plot freezing data from EXT1 and EXT2 on a single graph and apply appropriate statistical analyses to determine whether the sham group also demonstrates extinction

As recommended by the reviewer, we have performed an additional analysis comparing treatment groups by collapsing across trials (1-20) for each of the 3 extinction sessions using a 2-way ANOVA. This analysis revealed significant main effects for treatment ($p<.0001$) and session ($p<.0001$), but no significant session x treatment interaction. Post hoc analysis showed that rats in the THC withdrawal group exhibited significantly more freezing than Veh controls during Ext1 and Ext2 ($p<.0001$ for both sessions). Moreover, there was a significant decrease in freezing across sessions in both treatment groups (Veh, Ext1 vs. Ext2, $p<.0001$, Ext1 vs. Ext3, $p = 0.0456$; THC, Ext1 vs. Ext2, $p<.0001$, Ext1 vs. Ext3, $p<.0001$). Therefore, in answer to the reviewer's comment, both groups showed extinction across sessions, although the THC withdrawn rats exhibited more freezing during Ext1 and Ext2. This additional analysis is now included in the Supplement (Fig. S2) and described in results.

Normalizing freezing levels during the initial trials (e.g., trials 1–3) could provide additional clarity.

Moreover, a key concern arises in (1F), where sham mice do not exhibit freezing extinction in response to the CS.

It appears the reviewer is referring to the GCaMP8f signal data in Fig. 1F during extinction. As reported in the manuscript, there is a significant reduction or "extinction" of the GCaMP response to CS that was recorded from the rats.

-Freezing behavior should be shown longitudinally, from FC1 and FC2 through EXT1, EXT2, and the probe session.

Because we are interested in the effects of THC withdrawal on the effectiveness of fear conditioning and not its acquisition, and because of the difficulty in measuring freezing behavior during the conditioning process due to the presence of incompatible escape responses, we evaluated learning expression by measuring freezing to the CS only during the extinction trials; an analysis that is fairly common in the fear conditioning literature (e.g. (Nguyen et al., 2000; Poole et al., 2016; Rumpel et al., 2005).

We feel that combining the complete EXT1 and EXT2 sessions into a single data set is misleading because; 1. the extinction sessions occur on different days, and 2., because the freezing response is larger on the first trial block of EXT2 than that measured on the last trial block of EXT1. Therefore, when these 2 sessions are plotted together there is an increase in freezing on trial block 21-23 (the first block on day 2 of extinction) that appears artifactual. Therefore, to better demonstrate the reemergent freezing response to the CS on trial block 1 of the second extinction session, we feel it is better to show the results of EXT1 and EXT2 separately.

However, in line with the reviewer's reasoning, we have combined the sessions for purposes of statistical analysis and find that there is a significant main effect of trial in both groups, indicating that the freezing response diminishes as a function of the number of CS presentations. This is best indicated by the posthoc test showing that average freezing time differs for both treatment groups between Ext1 and Ext2. This is now described in the results section and the figure showing this analysis is included in the Supplement (Fig. S2).

-Although the LHb reliably responds to shocks, the z-score data for the CS (Supplementary Figure 1) are unclear as some mice in the sham group show no CS response.

The data to which the reviewer is referring in Fig. S1 shows GCaMP8f signals averaged across all rats and all trials (10) during the first fear conditioning session (FC1). The individual data points that are shown represent the average GCaMP8f signal acquired during CS presentation across all rats at each trial (1-10). The single smallest data point in the Veh treatment group represents the relative lack of response to the first presentation of CS. This is consistent with the absence of US-CS association prior to additional CS-US pairing trials. This information is now included in the revised figure caption (Fig. S1).

The authors should 1/ include picture(s) of GCaMP expression in the LHb to confirm consistent GCaMP expression and LHb targeting, and 2/ plot and test for the correlation between CS and US responses.

There is a representative image of GCaMP8f expression included in Fig.1, panel A.

We have performed the requested correlation between the CS- and US-associated calcium responses (see below) and find a weak but significant positive correlation between these variables. Although the reviewer does not state a purpose for performing this analysis, the data appear to show that smaller US calcium responses are correlated with smaller CS responses.

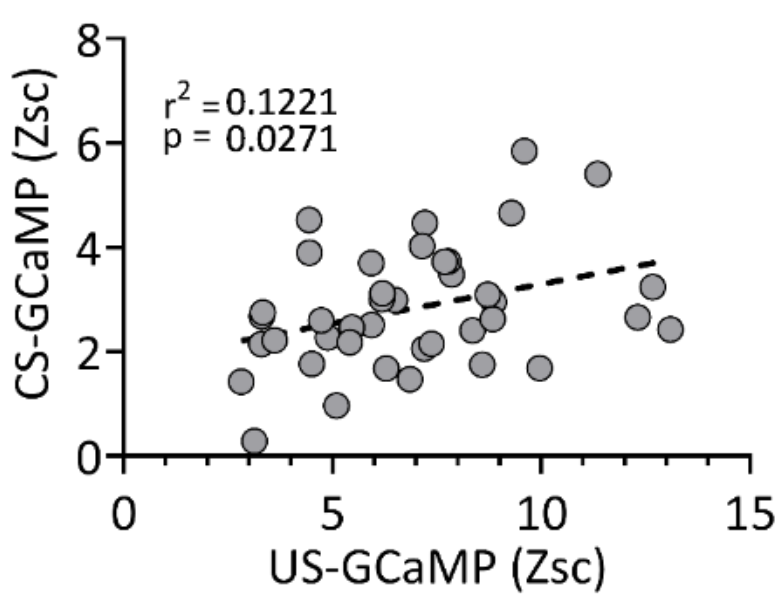

-In many instances, the largest differences appear during the initial trials. This may indicate a "startle response" rather than a genuine CS-US association Additional experiments with unpredictable shocks and CS tones will clarify this.

In examining the data, it is clear that the LHb calcium response to CS presentation increases in size in both treatment groups across the first 10 fear conditioning trials of FC1, with the THC group showing a significantly larger response to CS as the number CS-US associations increase (Fig. 1D). The observation that this CS-evoke increase occurs over several trials likely indicates that LHb neurons are encoding the CS-US association. If the differences between groups occurred because of an increase startle in the THC group this should be apparent during the first presentations of the CS during FC. Nevertheless, it is possible that during presentation of the CS alone during extinction that an increased sensitivity to the CS could result in increased freezing time in the THC withdrawal group.

To address this, we performed an additional experiment in which the CS tone is not explicitly paired with footshock. In this "unpaired experiment", during conditioning the footshock was delivered at random unpredictable intervals (20-90 sec) after the 10 sec CS presentation has ended. This was performed during 2 daily, 10-trial conditioning sessions, and the extent of conditioning was assessed

during extinction sessions 1-3 with CS presented alone. This experiment revealed that Veh and THC withdrawal group freezing times did not differ (no significant interaction between trial and treatment, nor main effects, 2-way ANOVA) suggesting that the significantly larger freezing response observed in our original "paired" CS-US experiment did not result from a heightened "startle response" to the CS. This experiment is now described in the Results and the data shown in Figure S4.

-Additionally, since LHb responses to shocks are stable over time in physiological condition but vary across trials, the authors must represent average responses across five trials (Figure 1D–E), rather than plotting trial-by-trial data.

Rather than dealing with the trial-by-trial variability by averaging every 5 trials we have decided to collapse these data across all trials and subjects within treatment conditions and perform unpaired t-tests on these data. This analysis is in addition to 2-way ANOVAs for the trial-by trial data reported in Results. The collapsed comparisons of GCaMP data are provided in supplemental Fig. S1.

FIGURE 2:

The data presented are compelling and well-executed, but a comprehensive understanding of withdrawal-induced plasticity requires chronic IPSC and EPSC recordings.

To achieve more robust statistical analysis, it is advisable to plot sIPSC and sEPSC frequency and amplitude as cumulative probability plots, employing methods such as the Kolmogorov-Smirnov (KS) test.

It is unclear what is meant by "chronic IPSC and EPSC recordings". These synaptic current experiments can only be performed in brain slices that are prepared acutely, and therefore cannot be done across time, as would be required by long-term chronic recordings.

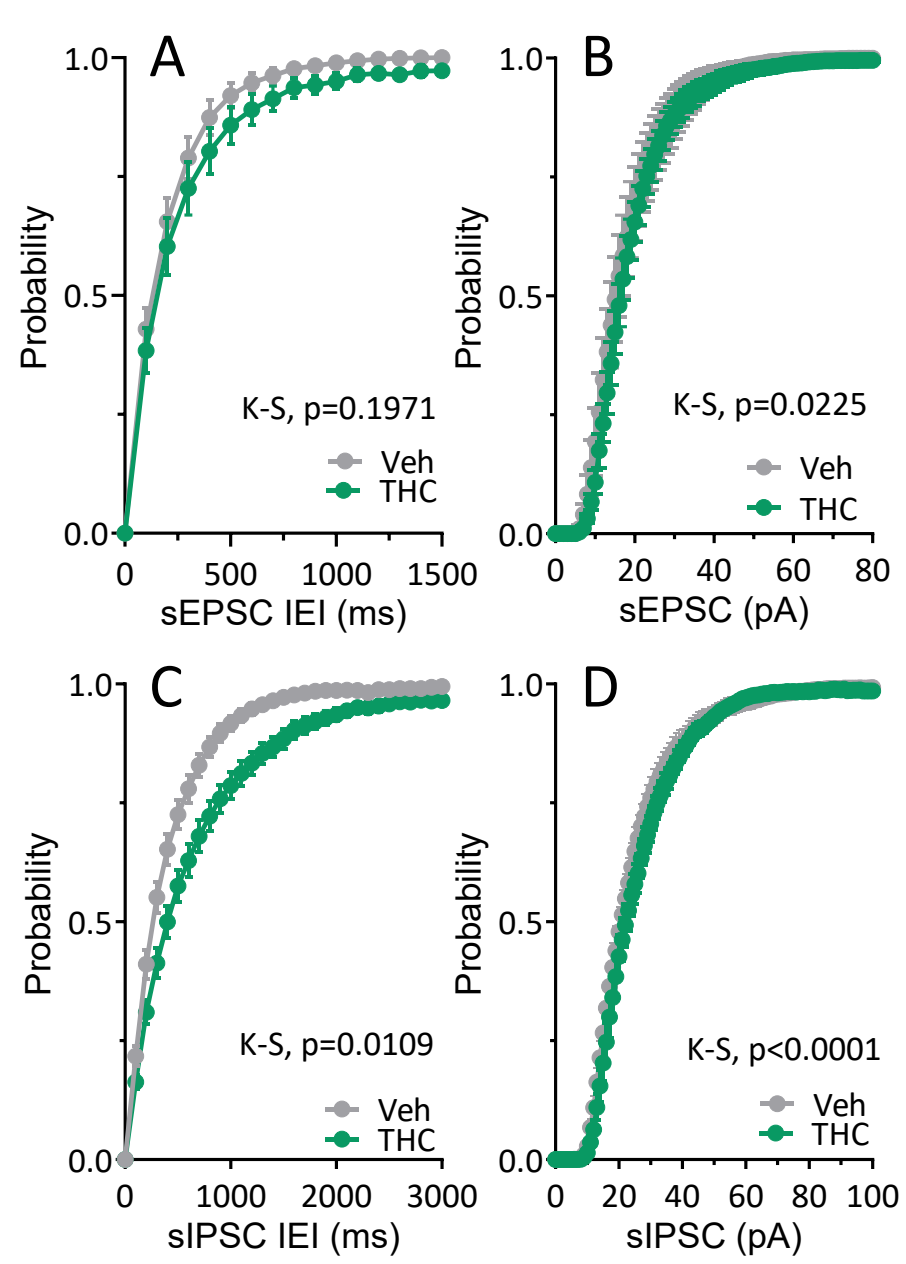

The K-S test is a non-parametric statistic that we feel is most useful to evaluate changes produced by within subject/cell treatments. In our experience it is not as useful for comparisons between groups because it fails to account for the inherent variability in synaptic current parameters across cells/subjects. Additionally, for analyses of spontaneous synaptic currents we find the K-S measure to be a very liberal statistical test that often reports significance where there is clearly no difference between distributions (e.g., left, panels B and D). Unfortunately, this also results in disagreements between results of the non-parametric K-S test applied to group data and parametric ANOVAs or t-tests applied to mean amplitude and frequency data. Because of these issues we usually restrict the use of K-S tests for within subjects studies and we utilize conservative p values (e.g., p <0.001) in evaluation of differences between distributions (Hoffman and Lupica, 2000; Lupica et al., 2001). Because of the shortcomings of the K-S test, we believe that it is statistically more rigorous for between groups data (such as in the present study) to compare treatment effects on mean

amplitudes and frequencies of synaptic currents that can be analyzed using parametric statistics such as ANOVAs. As reported in our manuscript, this approach shows that withdrawal from THC was associated with a decrease in the mean frequency of sIPSCs, and no changes in mean sIPSC amplitudes, and no changes in either mean sEPSC amplitudes or frequencies.

Because our experiment requires comparison between independent treatment groups, and because of the problems with K-S- test analysis of these data described above, we prefer to show mean amplitude and inter-event interval (IEI) data for spontaneous EPSCs and IPSCs. Additionally, the cumulative distributions for these groups that we show above demonstrate a significant K-S- test for an increase in sIPSC IEI that is consistent with our report of a decrease in GABA release probability in LHb neurons during THC withdrawal. However, the comparisons of spontaneous synaptic current amplitudes between these treatment groups reveals significant K-S tests, despite the clear absence of separation between the distributions. Therefore, to avoid confusing the reader we prefer not to show the results of the K-S tests and to only report mean frequencies and amplitudes in the manuscript.

There is concern regarding the withdrawal period selected for these experiments. Specifically, recordings were conducted one day after the cessation of THC administration, while in a previous figure, data were obtained two days post-withdrawal. This inconsistency raises questions about the reliability of the findings. How can one be confident that the excitatory-inhibitory imbalance remains as pronounced after 48 hours of withdrawal?

We agree with the reviewer that the timing of these synaptic analyses is important, and this was a primary reason why we performed experiments to define the duration of the synaptic impairment and CB1R desensitization during THC withdrawal. Thus, the results of these experiment shown in Fig. 4 indicate that the synaptic impairment was present on days 1-3 of withdrawal and did not fully recover up to 30 days. Therefore, as this impaired GABAergic inhibition of LHb neurons persist 1-30 days post withdrawal and underlies the increase in E-I ratio, it is likely E-I balance remained elevated at these latter time points.

Additionally, there is concern about how individual variability has been accounted for. The individual unit of analysis should be the rat rather than the recorded neuron. Therefore, all statistical analyses must be reevaluated to ensure methodological rigor and accurate interpretation of the data.

We appreciate and share the reviewer's concern for appropriate statistical and methodological rigor of scientific data. In all behavioral and calcium imaging experiments the unit of analysis was the rat. However, in the electrophysiological experiments the unit of analysis is the neuron, where 3-4 neurons per subject were included in the analyses. Additionally, the number of rats and neurons recorded for each of these experiments is reported in the figure captions and in the statistical summary supplement. Obtaining results from several neurons per subject in the electrophysiological experiments reduces the number of experimental animals and is a widely accepted approach for assessment of physiological measures in neurons. In all cases, the statistical analysis was chosen in accordance with the design of the experiment and largely relied upon ANOVAs, with appropriate post hoc analysis only when a significant ANOVA F statistic was observed.

FIGURE 3:

To fully assess the excitatory-inhibitory balance within LHb circuits, it is essential to conduct experiments measuring excitatory-inhibitory (E/I) ratios and paired-pulse ratios specifically for excitatory inputs.

The measurement of E-I ratios requires simultaneous measurement of excitatory and inhibitory synaptic input to cells. Therefore, recording E-I ratios specifically for excitatory inputs alone is not possible.

Our E-I ratio experiments were conducted using 2 methods. The first was to use electrical stimulation to simultaneously evoke inhibitory and excitatory synaptic currents. This showed a large significant increase in LHb E-I ratio in the THC withdrawn rats. Separate plots of these eEPSCs and eIPSCs across a range of stimulus intensities indicated that eEPSCs were unaffected during THC withdrawal. The second method was to record spontaneously occurring excitatory (sEPSCs) and inhibitory (sIPSCs) synaptic currents from LHb neurons in the absence of electrical stimulation. This experiment confirmed the reduction in GABA release probability in the absence of a change in glutamate release probability in the LHb of THC withdrawn rats. Moreover, only the frequency, and not the amplitude of sIPSCs was reduced, whereas neither frequency nor amplitude of sEPSCs was changed in the THC withdrawal LHb neurons. Therefore, the two approaches are entirely concordant. Given the complete agreement between these 2 measurements, and because both frequency and amplitude of glutamatergic currents were unaltered by THC exposure it is unlikely that release probability or postsynaptic sensitivity to glutamate was altered. Therefore, we feel that the use of paired pulse recordings of eEPSCs (an alternative measure of release probability) is redundant with our data.

One major challenge in studying the BF is its anatomical complexity, as it comprises multiple regions, including the ventral pallidum and nucleus accumbens. Research shows that many inputs to the LHb can release glutamate and GABA, either as corelease or from parallel inputs. Thus, targeting the BF is not precise enough. To determine whether the observed effects are consistent across BF's subregions, additional targeted experiments must be performed. Specifically, to identify the key input region responsible for LHb modulation, channelrhodopsins should be expressed selectively in either the VP or the nucleus accumbens.

We share the reviewer's concern for the anatomical delineation of the BF. To this end, we have provided extensive anatomical data to justify our inclusion of this brain region in our studies. These data indicate that this brain area provides anatomically verified input to the LHb that is comprised predominantly of GABAergic axons whose terminals express functional CB1Rs (Hwang et al., 2024).

In short, our choice of the particular BF location to target with channelrhodopsin-2 virus injections was based on our prior anatomical fluorogold retrograde tracing study confirming that neurons in this particular region of the BF provide GABAergic input to the LHb and express CB1Rs (Hwang et al., 2024). In addition, a nearly identical BF input to LHb has been implicated in social defeat reward (Golden et al., 2016). (Please see response to Reviewer #3).

The additional a more granular dissection of subnuclei to which the reviewer is referring would require an extensive number of experiments and likely require the use of mouse Cre driver lines. As we used rats in our studies, we feel that this would represent an extensive expansion of the project that is beyond the present scope.

Furthermore, employing two distinct opsins that respond to different wavelengths would allow for selective and simultaneous stimulation of both input pathways. This approach would enable researchers to test the hypothesis that effective LHb modulation requires cooperative input from both regions rather

than isolated activity from a single source. These experiments are crucial to refining our understanding of the network dynamics underlying LHb function during withdrawal states.

This is an excellent idea that will require extensive additional work that we would like to perform in future experiments.

Reviewer #2 (Remarks to the Author):

This is a highly compelling and meticulously executed study that significantly advances our understanding of the neurobiological underpinnings of cannabis withdrawal. The authors employ a sophisticated and complementary array of techniques—fiber photometry, in vitro electrophysiology, and ex vivo optogenetic experiments—to dissect the circuit-specific alterations in the lateral habenula (LHb) during Δ9-THC withdrawal in rats. Their findings provide robust and novel insights into how a dysregulation of endocannabinoid (eCB) signaling within specific GABAergic inputs to the LHb contributes to the negative affective states associated with cannabis withdrawal. The rigor of the experimental design and the clarity of the results make this a truly outstanding contribution to the field.

The central aim of this work was to unravel how cannabis withdrawal alters LHb function and its interactions with key GABAergic afferents, ultimately impacting fear-related behavior. Addressing a critical gap in the literature regarding the neurobiological consequences of cannabis withdrawal on brain function at the circuit level, the study provides strong evidence that Δ9-THC withdrawal leads to heightened LHb neuronal and population activity, which can directly contribute to increased behavioral sensitivity to aversive stimuli.

Specifically, the authors demonstrated using fiber photometry that withdrawn rats exhibited enhanced GCaMP calcium responses in the LHb during Pavlovian fear conditioning. This indicates a stronger encoding of aversive associations, consistent with the heightened negative affect observed during withdrawal from chronic cannabis use. This exaggerated activity was also observed during extinction sessions, concomitant with an increased freezing response to the conditioned stimulus, suggesting a persistent alteration in fear processing.

Mechanistically, the study precisely identified a selective impairment of inhibitory synaptic transmission within the LHb during withdrawal. Through in vitro electrophysiology, the authors demonstrated a significant reduction in the frequency of spontaneous inhibitory postsynaptic currents (sIPSCs) in LHb neurons from withdrawn rats. Crucially, this reduction was prevented by co-administration of the CB1 receptor (CB1R) antagonist AM251, strongly implicating the involvement of CB1Rs in this impairment of inhibitory control of LHb.

The ex vivo optogenetic experiments further refined this understanding, revealing that this GABAergic deficit was specific to inputs originating from the basal forebrain (BF), encompassing projections from the nucleus accumbens shell and ventral pallidum. In stark contrast, inhibitory inputs derived from the ventral tegmental area (VTA) to the LHb remained unaffected by Δ9-THC withdrawal. This input-specific dysregulation is a particularly significant finding, as it highlights a discrete circuit mechanism underlying the observed behavioral changes. The authors conclusively showed that the ability of presynaptic CB1Rs to inhibit GABA release is functionally compromised at BF inputs to the LHb for at least 30 days post-withdrawal, leading to a functional shift towards excitation and increased LHb excitability.

In sum, this study provides robust evidence that the negative affective state observed during cannabis

withdrawal is underpinned by a specific, long-lasting dysregulation of eCB modulation of GABAergic transmission from the basal forebrain to the LHb, resulting in an excitatory/inhibitory (E/I) imbalance within the LHb that can contribute to altered emotional processing and enhanced negative affect.

The study is exceptionally well-done and highly rigorous, providing compelling evidence for a novel mechanism in cannabis withdrawal.

We thank the reviewer for this positive assessment of our work.

I have only three minor comments for the authors to consider:

1. Methods Section Clarity (Electrophysiology & Optogenetics): I noted that the "Methods" section appears to lack specific subsections detailing the procedures for electrophysiological and optogenetic recordings. For the sake of clarity and reproducibility, it is crucial to include dedicated subsections outlining the precise protocols, equipment, software, and analysis methods used for both the in vitro electrophysiology and the ex vivo optogenetic experiments. This would enhance the manuscript's completeness and allow other researchers to replicate these technically challenging experiments.

To adhere to journal word limits, and because the electrophysiological and optogenetic methods that include all of these details are identical to a prior publication (Hwang et al., 2024), we provide this reference in the present manuscript Methods section.

2. Spontaneous Cell Firing Pattern Analysis: While the authors state that Δ9-THC withdrawal did not significantly alter the overall pattern of spontaneous cell firing (Fig. S1C, Chi-square value = 5.670, p = 0.1272), I am curious about the specifics of this analysis.

Could the authors clarify their criteria for classifying patterns of irregular versus regular tonic activity? A precise definition would be beneficial.

These designations were identical to those described in the classic studies of Rüdiger Veh (Wagner et al., 2017; Weiss and Veh, 2011). We now include this information in the Results section.

Furthermore, I am curious if combining the categories of "tonic" (both regular and irregular) and "bursting" cells and comparing this proportion to "silent" cells would reveal a significant shift in the proportion of spontaneously active versus silent neurons.

Given the profound changes in E/I balance and excitability observed elsewhere, a subtle shift in the overall proportion of active vs. silent cells might be masked by the current categorization, and exploring this alternative grouping could provide additional insight into the general excitability state of LHb neurons post-withdrawal.

We agree that a small shift in the excitability could be important. However, although post-hoc reclassification of neurons into merged categories violates recommendations for statistical rigor, we have collapsed the proportions as requested into 2 groups and reanalyzed using a Chi-Square test. This result remained non-significant (X^2 = 5.633, p = 0.06). Therefore, it appears that although there is a trend toward a reduction in the number of silent LHb neurons and a corresponding increase in bursting cells in the THC withdrawal group, this is not statistically significant. A sentence indicating this trend has been included in the Results.

3. Role of Astrocytes in LHb Hyperexcitability: The study provides compelling evidence for neuronal mechanisms underlying LHb hyperexcitability. However, the rich literature on eCB-regulation of astrocytes, particularly within the LHb, suggests that these glial cells could also contribute to the heightened LHb activity observed during cannabis withdrawal. Given that CB1Rs are expressed in LHb astrocytes and their activation can modulate synaptic transmission and calcium signaling as well as negative affective states, a discussion of this possibility would be a valuable addition. Considering how astrocytic eCB signaling might be altered during withdrawal and subsequently influence neuronal function could provide a more comprehensive picture of the circuit-level changes.

For calcium measurements, we used AAV-syn-jGCaMP8f-WPRE which used a synapsin promoter to drive GCaMP8f expression in neurons rather than astrocytes. Therefore, it is unlikely that our in vivo imaging sampled calcium signals from astrocytes. As for the effect of activation of astrocytic CB1Rs in the LHb, this appears to indirectly facilitate synaptic glutamate release via an unknown mechanism (Winters et al., 2023), and our study showed that synaptic glutamate release was unchanged during Δ^9-THC withdrawal. It is possible that one of the acute effects of cannabinoids is to stimulate glutamate release from astrocytes via activation of astrocytic CB1Rs, and this effect may be desensitized during Δ^9-THC treatment. However, since we sampled LHb glutamatergic synapses only during Δ^9-THC withdrawal and not in the presence of CB1R agonists our data do not permit us to more directly address this possibility. We have added 2 sentences to the Discussion to address this possibility.

Reviewer #3 (Remarks to the Author):

In this work, the authors put forward the hypothesis that the lateral habenula contributes to aspects of cannabis withdrawal. Experimentally, the authors evaluated this by mean of photometric measurement of calcium responses during fear conditions in rats that experienced withdrawal from Δ9-THC. The main finding is that aversive footshock and predictive CS lead to GCaMP8f signal increases in withdrawal compared to vehicle animals. This higher calcium dynamics was linked to increased freezing during CS presentation after cannabis vapor exposure. Using slice physiology, the authors reported changes in E/I balance stemming from reduced synaptic inhibition of LHb neurons – these effects were CB1R dependent using specific pharmacology.

This study expands on the notion that plastic adaptations in the lateral habenula underscore aspects of drug withdrawal and bring new insights on cannabis withdrawal which at the moment was lacking. The combination of different approaches is elegant and the question is timely and certainly of interest for the field.

We thank the reviewer for the positive assessment of our work.

Major concerns.
1. The calcium dynamics elicited after shock and CS raise some question. The representative traces indicate a rundown across trials. This is visible in the Ext sessions and partly also in the FC sessions.

Photobleaching occurs with most fluorescent proteins. Fortunately, this process has predictable kinetics that can be compensated by subtracting the photobleaching time course from the signal baseline. Additionally, the influence of photobleaching is minimized by measuring the transient (US or CS-evoked) signal relative to baseline using the ΔF/F convention, prior to conversion of the signals to Z-

scores (equal to the number of standard deviations from the mean). This signal processing is performed by the Synapse software, and our data have been corrected for these changes.

Furthermore, it is unclear why only CS trial 4 and 6 are larger. How is the distribution of responses across mice? Do the authors see systematically such CS driven larger transients only on these trials? This should be further quantified and clarified in the text.

As required by the experimental design, these data were analyzed using 2-way ANOVAs that indicate significant treatment group x trial interactions and a significant main effect of treatment. This indicates overall significant differences between groups with regard to LHb neuron responses to the US and to CS presentation. The absence of a significant post-hoc test on every trial is secondary to the overall significant ANOVA. However, this may reflect the inherent variability associated with the sampling interval, as well as variability between and within subject. One way to deal with the trial-to-trial variability is to present the same data collapsed across all trials as an additional analysis (e.g., Fig. S1). This has now been included for the GCaMP data in FC1, FC2 and 3 extinction sessions in Fig. S1. Additionally, the collapsed freezing data across extinction sessions 1-3 are also now presented (Fig. S2). In Fig. S1A-C we report a significant increase in GCaMP8f signal to the CS (1 sample t-test) during FC1 and FC2 sessions. Moreover, we also show in Fig. S1A that treatment groups differ with regard to the size of the signal increase (unpaired t-test). Although these collapsed data reduce variability we feel that it remains necessary to show the trial-to-trial variability to provide a more honest depiction of the data as provided in Fig. 1.

When the authors assess freezing behaviors in panel K, freezing seems augmented only on certain trials, but during those trials CS dynamics in LHb seems not really altered.

The authors should provide some discussion around this point and if possible a manipulation of the circuit to understand the link between function and behavior.

Upon reanalysis of the data with new subjects added (Fig. 1K), we find that there are no significant differences between treatment groups on the freezing measure in Ext3 (i.e., no treatment x trial interaction or main effects by 2-way ANOVA). Similarly, significant effects were not observed in the GCaMP signal during the probe test (Ext3, Fig. 1H). This is also consistent with Fig. S1F and Fig. S2 where the GCaMP signal and freezing time during Ext3 were not significant when the data were collapsed across trials and subjects. Therefore, although it appears that the calcium signal is larger in the first trial block in THC withdrawn rats (Fig. 1H) and the difference in freezing is close to statistical significance, we cannot claim that the groups are different on these measures during Ext3. This has been revised in the results section.

2. The question on whether such activity dynamics plasticity in fear conditioning is a feature of cannabis withdrawal or encompasses other drugs should be tested.

Now that we have provided evidence that the LHb is likely involved in drug withdrawal, the possible generalizability to other modeled use disorders is of course an important future direction. With reference to opioid actions, mu opioid receptors are present in the LHb, and there is evidence that LHb neurons track the aversive maintenance phase of fentanyl self-administration (Bailly et al., 2023; Coffey et al., 2023). Therefore, we have planned experiments similar to those presented in the manuscript in opioid withdrawn rats. Additionally, as we describe in the manuscript, there is evidence suggesting that cocaine withdrawal is associated with impaired entopeduncular nucleus GABAergic

input to the LHb, which would also be expected to contribute to LHb hyperexcitability and aversion (Meye et al., 2016). However, the behavioral, photometry, and electrophysiological studies required for rigorous comparisons among drug classes are in our opinion best reserved for another study.

3. The authors discuss the importance of basal forebrain inputs onto LHb mentioning regions of the pallidum and accumbens. However it remains unclear where these axonal innervation comes from.

In addition the lack of injections site visualisation and innervation in the habenula is lacking. This portion of the study deserves a much thorough dissection to define the inputs organisation as BF remains a vague term encompassing large territories with different behavioral relevance.

We thank the reviewer for allowing us to clarify this point. Indeed, much of this information is contained in our prior publication that is cited in reference to these points in the present manuscript (Hwang et al., 2024). In this prior work we identified neurons projecting to the LHb using retrograde fluorogold immunoreactivity (FG-IR), that was co-localized with CB1R and glutamic acid decarboxylase (GAD) mRNA. A large number of these neurons are located in the area that we refer to as the BF are found in specific regions of the VP and NAc shell, and to a lesser extent in portions of the horizontal diagonal band of Broca (HDB). These anatomical regions are considered part of the BF. As such, we have defined this projection based on the colocalization of these markers after specific FG injection into the LHb rather than using other anatomical boundaries. More specifically, we found that, FG-IR was densest in caudal regions of the NAc shell and in the most rostral region of the VP (Hwang et al., 2024). Thus, our rationale for localizing ChR2 injections to this specific region is based upon the previous observation that it projects to the LHb, is GABAergic, and is modulated by exogenous and endogenous cannabinoids.

Moreover, although the VP is often thought of as a homogenous functional unit, it is in fact a large neuroanatomical structure that is heterogeneous with regard to its projection targets and cellular phenotypes (Bernat et al., 2024; Faget et al., 2018; Kupchik and Prasad, 2021). Our ChR2-virus injections specifically target the rostral VP, where we observed colocalization of FG-IR, and CB1 and GAD mRNA in neurons projecting to the LHb (Hwang et al., 2024). This rostral VP region contains a much smaller proportion of neurons expressing vesicular glutamate transporter (VGluT2) mRNA, compared to the caudal VP (Faget et al., 2018). In addition to this cellular heterogeneity within the area collectively known as the VP, there is also significant target-dependent behavioral heterogeneity of VP projections (Bernat et al., 2024; Golden et al., 2016; Knowland et al., 2017; Kupchik and Prasad, 2021; Leung and Balleine, 2015). Of particular interest, the same region of VP projecting to LHb that we identified using retrograde tracing has also been implicated in social defeat reward, where it is also referred to as a BF projection to the LHb (Golden et al., 2016). Therefore, this prior study provides an anatomical precedent for the nomenclature that we use.

We do agree with the reviewer that the sites of viral injections within the BF used in the present study are important to address. However, because the ex-vivo experiments required that the brains of the rats receiving virus injections be dissected to produce slices containing the LHb, we do not have histology for each subject. Instead, throughout these studies, and within each cohort, we performed routine histological verification of the locations of these injections. Schematic reconstructions of these representative injections obtained from 16 rats are now provided in the supplement (Fig. S6). Additionally, in a previous publication we show representative ChR2-associated eYFP expression in the LHb 8 weeks after similar injections in the BF (Hwang et al., 2024), and in all ChR2 electrophysiology experiments in the present study, eYFP expression was confirmed in each brain slice prior to patching

neurons in the LHb. Although we do collect these images using video microscopy on our electrophysiology rigs, they are used to confirm expression and are not suitable for publication.

4. The comparison between BF and VTA is interesting. However, a rationale for choosing the VTA remains elusive. GABA inputs onto the habenula emerge from the GPi in the basal ganglia, as well as the septum. Is a comparison limited to the VTA appropriate? The authors should experimentally test this concern.

The VTA input to LHb was chosen because of our prior experience with this projection (Root et al., 2014), and because its cannabinoid sensitivity was previously unknown. The GPi, or the entopeduncular nucleus input to LHb in rodents, has been shown to be insensitive to cannabinoids (Winters et al., 2023). We are planning to explore the behavioral relevance of other LHb afferents, including the medial septal nucleus, in future studies.

5. Presynaptic GABA release is controlled by different machineries. One is the endocannabinoid system, but others relie on GABAb receptors or opioid receptors. The authors could test some controls to understand if other machineries adapt as well during cannabis withdrawal.

In the course of these studies, we examined whether the selective mu opioid receptor (MOR) agonist DAMGO altered oIPSCs evoked by photostimulation of BF inputs to LHb during withdrawal from THC. We observed a peak inhibition of oIPSCs (26 ± 4.4%) by DAMGO (1 µM) in the Veh withdrawn group ($n = 7$ neurons). However, this peak inhibition did not differ from that observed in LHb neurons from THC withdrawn rats (25± 8.9% inhibition, $n = 6$; $t=0.066$, $df=11$, $p = 0.95$). Curiously, unlike our experiments with CB1R agonists, reversal of the DAMGO effect was incomplete with extended 30 min washouts. Thus, the data are more complex and perhaps indicate that DAMGO initiated a form of long-term synaptic plasticity that will require more extensive characterization. Therefore, although comparisons of the effects of additional GPCRs on BF-LHb inputs during THC withdrawal will be required, our data suggest that, at least in comparison to mu opioid receptors, long-term THC exposure selectively disrupts CB1R modulation of GABA release. Because of the complexity of these opioid receptor data we would prefer to more completely examine overlap to other presynaptic GPCRs in future studies.

References

Bailly, J., Allain, F., Schwartz, E., Tirel, C., Dupuy, C., Petit, F., Diana, M.A., Darcq, E., Kieffer, B.L., 2023. Habenular Neurons Expressing Mu Opioid Receptors Promote Negative Affect in a Projection-Specific Manner. Biol Psychiatry 93, 1108–1117.

Bernat, N., Campbell, R.R., Nam, H., Basu, M., Odesser, T., Elyasaf, G., Engeln, M., Chandra, R., Golden, S., Ament, S., Lobo, M.K., Kupchik, Y.M., 2024. Multimodal Interrogation of Ventral Pallidum Projections Reveals Projection-Specific Signatures and Effects on Cocaine Reward. J Neurosci 44.

Burston, J.J., Wiley, J.L., Craig, A.A., Selley, D.E., Sim-Selley, L.J., 2010. Regional enhancement of cannabinoid CB1 receptor desensitization in female adolescent rats following repeated Delta-tetrahydrocannabinol exposure. Br J Pharmacol 161, 103–112.

Coffey, K.R., Nickelson, W.B., Dawkins, A.J., Neumaier, J.F., 2023. Rapid appearance of negative emotion during oral fentanyl self-administration in male and female rats. Addict Biol 28, e13344.

Faget, L., Zell, V., Souter, E., McPherson, A., Ressler, R., Gutierrez-Reed, N., Yoo, J.H., Dulcis, D., Hnasko, T.S., 2018. Opponent control of behavioral reinforcement by inhibitory and excitatory projections from the ventral pallidum. Nat Commun 9, 849.

Farquhar, C.E., Breivogel, C.S., Gamage, T.F., Gay, E.A., Thomas, B.F., Craft, R.M., Wiley, J.L., 2019. Sex, THC, and hormones: Effects on density and sensitivity of CB(1) cannabinoid receptors in rats. Drug Alcohol Depend 194, 20–27.

Golden, S.A., Heshmati, M., Flanigan, M., Christoffel, D.J., Guise, K., Pfau, M.L., Aleyasin, H., Menard, C., Zhang, H., Hodes, G.E., Bregman, D., Khibnik, L., Tai, J., Rebusi, N., Krawitz, B., Chaudhury, D., Walsh, J.J., Han, M.-H., Shapiro, M.L., Russo, S.J., 2016. Basal forebrain projections to the lateral habenula modulate aggression reward. Nature 534, 688–692.

Hoffman, A.F., Lupica, C.R., 2000. Mechanisms of cannabinoid inhibition of GABA(A) synaptic transmission in the hippocampus. J Neurosci 20, 2470–2479.

Hoffman, A.F., Oz, M., Yang, R., Lichtman, A.H., Lupica, C.R., 2007. Opposing actions of chronic D9-tetrahydrocannabinol and cannabinoid antagonists on hippocampal long-term potentiation. Learn. Mem 14, 63–74.

Huestis, M.A., Henningfield, J.E., Cone, E.J., 1992. Blood cannabinoids. I. Absorption of THC and formation of 11-OH-THC and THCCOOH during and after smoking marijuana. J Anal Toxicol 16, 276–282.

Hwang, E.K., Zapata, A., Hu, V., Hoffman, A.F., Wang, H.L., Liu, B., Morales, M., Lupica, C.R., 2024. Basal forebrain-lateral habenula inputs and control of impulsive behavior. Neuropsychopharmacology 49, 2060–2068.

Knowland, D., Lilascharoen, V., Pacia, C.P., Shin, S., Wang, E.H., Lim, B.K., 2017. Distinct Ventral Pallidal Neural Populations Mediate Separate Symptoms of Depression. Cell 170, 284–297 e218.

Kupchik, Y.M., Prasad, A.A., 2021. Ventral pallidum cellular and pathway specificity in drug seeking. Neurosci Biobehav Rev 131, 373–386.

Leung, B.K., Balleine, B.W., 2015. Ventral pallidal projections to mediodorsal thalamus and ventral tegmental area play distinct roles in outcome-specific Pavlovian-instrumental transfer. J Neurosci 35, 4953–4964.

Lupica, C.R., Bell, J.A., Hoffman, A.F., Watson, P.L., 2001. Contribution of the hyperpolarization-activated current (Ih) to membrane potential and GABA release in hippocampal interneurons. J. Neurophysiol 86, 261–268.

McLaren, J., Swift, W., Dillon, P., Allsop, S., 2008. Cannabis potency and contamination: a review of the literature. Addiction 103, 1100–1109.

Meye, F.J., Soiza-Reilly, M., Smit, T., Diana, M.A., Schwarz, M.K., Mameli, M., 2016. Shifted pallidal co-release of GABA and glutamate in habenula drives cocaine withdrawal and relapse. Nat Neurosci 19, 1019–1024.

Nguyen, J.D., Aarde, S.M., Vandewater, S.A., Grant, Y., Stouffer, D.G., Parsons, L.H., Cole, M., Taffe, M.A., 2016. Inhaled delivery of Delta(9)-tetrahydrocannabinol (THC) to rats by e-cigarette vapor technology. Neuropharmacology 109, 112–120.

Nguyen, P.V., Abel, T., Kandel, E.R., Bourtchouladze, R., 2000. Strain-dependent differences in LTP and hippocampus-dependent memory in inbred mice. Learn Mem 7, 170–179.

Poole, R.L., Braak, D., Gould, T.J., 2016. Concentration- and age-dependent effects of chronic caffeine on contextual fear conditioning in C57BL/6J mice. Behav Brain Res 298, 69–77.

Root, D.H., Mejias-Aponte, C.A., Zhang, S., Wang, H.L., Hoffman, A.F., Lupica, C.R., Morales, M., 2014. Single rodent mesohabenular axons release glutamate and GABA. Nat Neurosci 17, 1543–1551.

Rubino, T., Vigano, D., Realini, N., Guidali, C., Braida, D., Capurro, V., Castiglioni, C., Cherubino, F., Romualdi, P., Candeletti, S., Sala, M., Parolaro, D., 2008. Chronic delta 9-tetrahydrocannabinol during adolescence provokes sex-dependent changes in the emotional profile in adult rats: behavioral and biochemical correlates. Neuropsychopharmacology 33, 2760–2771.

Rumpel, S., LeDoux, J., Zador, A., Malinow, R., 2005. Postsynaptic receptor trafficking underlying a form of associative learning. Science 308, 83–88.

Sim-Selley, L.J., 2003. Regulation of cannabinoid CB1 receptors in the central nervous system by chronic cannabinoids. Crit Rev. Neurobiol 15, 91–119.

Wagner, F., Weiss, T., Veh, R.W., 2017. Electrophysiological properties of neurons and synapses in the lateral habenular complex (LHb). Pharmacol Biochem Behav 162, 38–45.

Weiss, T., Veh, R.W., 2011. Morphological and electrophysiological characteristics of neurons within identified subnuclei of the lateral habenula in rat brain slices. Neuroscience 172, 74–93.

Winters, N.D., Kondev, V., Loomba, N., Delpire, E., Grueter, B.A., Patel, S., 2023. Opposing retrograde and astrocyte-dependent endocannabinoid signaling mechanisms regulate lateral habenula synaptic transmission. Cell Rep 42, 112159.

Response to REVIEWERS' COMMENTS on revision

Reviewer #1 (Remarks to the Author):

The authors have adequately and thoroughly addressed my concerns. The additional experiments and analyses are greatly appreciated. Congratulations on a job well done.

Reviewer #2 (Remarks to the Author):

The authors have comprehensively addressed all of the issues raised in my previous assessment, and I have no further comments. The revised manuscript is excellent. This work is highly significant, and I congratulate the authors for accomplishing such an outstanding study, particularly given the substantial difficulties they endured during the government shutdown.

Reviewer #3 (Remarks to the Author):

The authors have addressed my concerns. The outcome of this work is of excellent quality further supporting evidence for the role of habenular adaptations in drug withdrawal processes. I support publication in its current form.

We would like to express our gratitude to the reviewers for taking the time to review our manuscript and for their contributions in helping us improve our work.
