# Supplementary material for: Increased encoding of aversive stimuli by lateral habenula neurons during Δ9-tetrahydrocannabinol withdrawal
Source: Nat Commun. 2026 Sep 3;17:10485. doi: 10.1038/s41467-026-77429-9 (PMC13635186; doi:10.1038/s41467-026-77429-9)
Supplement: Supplementary file 3 [file 41467_2026_77429_MOESM3_ESM.pdf]

Description of Additional Supplementary Files

Supplementary Data 1: Statistical analysis summary
