# Supplementary material for: Increased encoding of aversive stimuli by lateral habenula neurons during Δ9-tetrahydrocannabinol withdrawal
Source: Nat Commun. 2026 Sep 3;17:10485. doi: 10.1038/s41467-026-77429-9 (PMC13635186; doi:10.1038/s41467-026-77429-9)
Supplement: Supplementary file 4 [file 41467_2026_77429_MOESM5_ESM.pdf]

nature portfolio

Corresponding author(s): Carl Lupica, Ph.D.

Last updated by author(s): August 11, 2026

Reporting Summary

Nature Portfolio wishes to improve the reproducibility of the work that we publish. This form provides structure for consistency and transparency in reporting. For further information on Nature Portfolio policies, see our Editorial Policies and the Editorial Policy Checklist.

Statistics

For all statistical analyses, confirm that the following items are present in the figure legend, table legend, main text, or Methods section.

n/a	Confirmed	
☐	☒	The exact sample size (*n*) for each experimental group/condition, given as a discrete number and unit of measurement
☐	☒	A statement on whether measurements were taken from distinct samples or whether the same sample was measured repeatedly
☐	☒	The statistical test(s) used AND whether they are one- or two-sided<br>*Only common tests should be described solely by name; describe more complex techniques in the Methods section.*
☒	☐	A description of all covariates tested
☐	☒	A description of any assumptions or corrections, such as tests of normality and adjustment for multiple comparisons
☐	☒	A full description of the statistical parameters including central tendency (e.g. means) or other basic estimates (e.g. regression coefficient) AND variation (e.g. standard deviation) or associated estimates of uncertainty (e.g. confidence intervals)
☐	☒	For null hypothesis testing, the test statistic (e.g. *F*, *t*, *r*) with confidence intervals, effect sizes, degrees of freedom and *P* value noted<br>*Give P values as exact values whenever suitable.*
☒	☐	For Bayesian analysis, information on the choice of priors and Markov chain Monte Carlo settings
☒	☐	For hierarchical and complex designs, identification of the appropriate level for tests and full reporting of outcomes
☒	☐	Estimates of effect sizes (e.g. Cohen's *d*, Pearson's *r*), indicating how they were calculated

Our web collection on statistics for biologists contains articles on many of the points above.

Software and code

Policy information about availability of computer code

Data collection	Fiber Photometry: Synapse Software v102-55329, Tucker -Davis Technologies (https://www.tdt.com/product/synapse-software/). Electrophysiology: Clampfit v9.0 (Molecular Devices), Win EDR v4.1.7 (University of Strathclyde, Glasgow, UK), WinLTP software v1.11 (WinLTP Ltd; www.winltp.com). Behavior: ANY-Maze software v7.67 (Stoelting, Wood Dale, Il, USA).
Data analysis	Fiber Photometry: Photometry Modular Analysis Tool, pMAT v1.2 (https://github.com/djamesbarker/pMAT). Electrophysiology: Clampfit v9.0 (Molecular Devices), Win EDR v4.1.7 (University of Strathclyde, Glasgow, UK). Behavior: ANY-Maze software (Stoelting, Wood Dale, Il, USA). Statistics and graphing: GraphPad Prism, v11.0 (Boston, MA, USA).

For manuscripts utilizing custom algorithms or software that are central to the research but not yet described in published literature, software must be made available to editors and reviewers. We strongly encourage code deposition in a community repository (e.g. GitHub). See the Nature Portfolio guidelines for submitting code & software for further information.

Data

Policy information about availability of data

All manuscripts must include a data availability statement. This statement should provide the following information, where applicable:

- Accession codes, unique identifiers, or web links for publicly available datasets
- A description of any restrictions on data availability
- For clinical datasets or third party data, please ensure that the statement adheres to our policy

The data described in this study are available as Source Data associated with this publication.

Research involving human participants, their data, or biological material

Policy information about studies with human participants or human data. See also policy information about sex, gender (identity/presentation), and sexual orientation and race, ethnicity and racism.

Reporting on sex and gender	N/A
Reporting on race, ethnicity, or other socially relevant groupings	N/A
Population characteristics	N/A
Recruitment	N/A
Ethics oversight	N/A

Note that full information on the approval of the study protocol must also be provided in the manuscript.

Field-specific reporting

Please select the one below that is the best fit for your research. If you are not sure, read the appropriate sections before making your selection.

☐ Life sciences ☒ Behavioural & social sciences ☐ Ecological, evolutionary & environmental sciences

For a reference copy of the document with all sections, see nature.com/documents/nr-reporting-summary-flat.pdf

Behavioural & social sciences study design

All studies must disclose on these points even when the disclosure is negative.

Study description	The study utilized 3 distinct data sets. One a behavioral assessment of freezing behavior, another from in vivo measurement of brain calcium signals, and the 3rd, an assessment of synaptic properties in vitro. Each data set used random assignment to experimental groups and using a between subjects design throughout. All of the animals used in fiber photometry experiments were included in the behavioral paired-conditioning portion of the study. Additional non-implanted rats were added to assess fear conditioning behavioral extinction as well as to power an analysis of sex-dependent differences in behavior.
Research sample	Wildtype Long-Evans Rats were used for all experiments. Males and females, all between 42 and 72 days of age at the beginning of procedures) were included in the behavioral and calcium measurement study. Only males were included in the electrophysiological portion of the study. These subjects were chosen based on compatibility with standard laboratory paradigms and procedures.
Sampling strategy	Rats were randomly assigned to treatment groups after recovery from surgery and testing for calcium signals using photometry. For electrophysiology, experiments were designed using estimates of effect size and dispersion obtained from experience with similar LHb brain slice preparations. These estimates were then entered into power analysis calculations using G-Power (version 3.1.9.7, University of Dusseldorf, Germany). Behavioral experiments also used G-Power to estimate sample size based on expected effect magnitudes.
Data collection	Fiber photometry data were collected using an RZ10x system (Tucker-Davis Technologies, Alachua, FL). Electrophysiological data were collected using gradient contrast microscopy to visually identify neurons in brain slices. Electrophysiological data were obtained using Multiclamp 700B amplifier (Molecular Devices, San Jose, CA), WinLTP software (WinLTP Ltd; www.winltp.com), and an A/D board (National Instruments PCI-6251, Austin, TX) residing in a personal computer. Researchers were not blinded to experimental condition or hypothesis because the researcher collecting the data was also responsible for assignment to experimental groups.
Timing	Experiments began in June 2022 and ended May, 2026.
Data exclusions	Pre-exclusion criteria were only used in the fiber photometry experiments. Rats were excluded from the experiment if calcium signals were not apparent, if calcium signals did not increase during air puff test, or if calcium signals degraded during the fear conditioning and extinction procedures. Eleven of 30 implanted rats were excluded based on these criteria. In some cases spurious data points caused by electrical interference (electrophysiology) or a disconnected head mounts (fiber photometry) were excluded from analysis.
Non-participation	N/A
Randomization	All experiments used random assignment to experimental groups.

Reporting for specific materials, systems and methods

We require information from authors about some types of materials, experimental systems and methods used in many studies. Here, indicate whether each material, system or method listed is relevant to your study. If you are not sure if a list item applies to your research, read the appropriate section before selecting a response.

Materials & experimental systems

n/a	Involved in the study
[ ]	[x] Antibodies
[x]	[ ] Eukaryotic cell lines
[x]	[ ] Palaeontology and archaeology
[ ]	[x] Animals and other organisms
[x]	[ ] Clinical data
[x]	[ ] Dual use research of concern
[x]	[ ] Plants

Methods

n/a	Involved in the study
[x]	[ ] ChIP-seq
[x]	[ ] Flow cytometry
[x]	[ ] MRI-based neuroimaging

Antibodies

Antibodies used: mouse anti-GFP-632381, 1:1000, mouse (Takara Bio, USA), Alexa Fluor 488, donkey anti-mouse, 715545151, Jackson Laboratories, USA

Validation: validation was performed by comparison with nearby tissue where GFP was not virally expressed.

Animals and other research organisms

Policy information about studies involving animals; ARRIVE guidelines recommended for reporting animal research, and Sex and Gender in Research

Laboratory animals: Long-Evans rats 42-72 days old.

Wild animals: N/A

Reporting on sex: Sex-based analysis was used for behavioral freezing data (n = 18), and no differences between sexes were observed using Two-way ANOVA, or unpaired 2 tail t-tests. Sex-based analyses were not performed for fiber photometry because the study did not have sufficient numbers to power this analysis and because there were no sex-related differences in freezing behavior. The "unpaired" conditioning experiment used only males because no differences were observed in freezing behavior between males and females in the paired conditioning experiments. The electrophysiological studies used only male rats because several studies report nearly identical changes in CB1R function males and females after long-term THC treatment.

Field-collected samples: N/A

Ethics oversight: The animal procedures were approved by the NIDA-IRP Animal Care and Use Committee for ethical considerations. The NIDA-IRP animal facility is accredited by Association for Assessment and Accreditation of Laboratory Animal Care (AAALAC) International.

Note that full information on the approval of the study protocol must also be provided in the manuscript.

Plants

Seed stocks: N/A

Novel plant genotypes: N/A

Authentication: N/A
